# Major Oncogenic Drivers and Their Clinicopathological Correlations in Sporadic Childhood Papillary Thyroid Carcinoma in Belarus

**DOI:** 10.3390/cancers13133374

**Published:** 2021-07-05

**Authors:** Tatiana I. Rogounovitch, Svetlana V. Mankovskaya, Mikhail V. Fridman, Tatiana A. Leonova, Victor A. Kondratovitch, Natalya E. Konoplya, Shunichi Yamashita, Norisato Mitsutake, Vladimir A. Saenko

**Affiliations:** 1 Department of Radiation Medical Sciences, Atomic Bomb Disease Institute, Nagasaki University, Nagasaki 852-8523, Japan; tatiana@nagasaki-u.ac.jp (T.I.R.); mitsu@nagasaki-u.ac.jp (N.M.); 2 Institute of Physiology of the National Academy of Sciences of Belarus, 220072 Minsk, Belarus; mankovskaya_svet@mail.ru; 3 Republican Centre for Thyroid Tumors, Department of Pathology, Minsk City Clinical Oncologic Dispensary, 220013 Minsk, Belarus; mfridman@tut.by; 4 Counseling-Diagnostic Department of Thyroid Diseases, Minsk City Clinical Oncologic Dispensary, 220013 Minsk, Belarus; avinir@mail.ru; 5 Minsk City Clinical Oncologic Dispensary, 220013 Minsk, Belarus; mgkod@mail.ru; 6 N.N.Alexandrov National Cancer Centre of Belarus, Department of Chemotherapy, 223040 Minsk, Belarus; nkonoplya@mail.ru; 7 Radiation Medical Science Center, Fukushima Medical University, Fukushima 960-1295, Japan; shun@nagasaki-u.ac.jp; 8 Center for Advanced Radiation Emergency Medicine, National Institutes for Quantum and Radiological Science and Technology, Chiba 263-8555, Japan; 9 Department of Radiation Molecular Epidemiology, Atomic Bomb Disease Institute, Nagasaki University, Nagasaki 852-8523, Japan

**Keywords:** papillary thyroid carcinoma, sporadic childhood thyroid cancer, Chernobyl, mutation, gene rearrangement, clinicopathological characteristics

## Abstract

**Simple Summary:**

Childhood papillary thyroid carcinomas (PTCs) detected after the Chernobyl accident were genetically characterized by a high prevalence of gene rearrangements and low frequency of point mutations. However, no reports on genetic alterations in sporadic childhood PTCs from the Chernobyl-affected regions are available. This study investigated a series of PTCs from children of Belarus not exposed to radiation, and found that fusion genes were significantly more prevalent than point mutations in these tumors. Clinicopathologically, *RET/PTC3* was associated with solid growth pattern and higher tumor aggressiveness, *BRAF^V600E^* and *RET/PTC1* with classic papillary morphology and mild clinical phenotype, and *ETV6ex4/NTRK3* with follicular-patterned PTC and the reduced aggressiveness. The spectrum of driver mutations in sporadic childhood PTC largely parallels that in Chernobyl PTC, although the distribution of oncogene types suggests less aggressive clinical presentation of sporadic PTC, especially than that of early-onset radiation-related PTC.

**Abstract:**

Childhood papillary thyroid carcinoma (PTC) diagnosed after the Chernobyl accident in Belarus displayed a high frequency of gene rearrangements and low frequency of point mutations. Since 2001, only sporadic thyroid cancer occurs in children aged up to 14 years but its molecular characteristics have not been reported. Here, we determine the major oncogenic events in PTC from non-exposed Belarusian children and assess their clinicopathological correlations. Among the 34 tumors, 23 (67.6%) harbored one of the mutually exclusive oncogenes: 5 (14.7%) *BRAF^V600E^*, 4 (11.8%) *RET/PTC1*, 6 (17.6%) *RET/PTC3*, 2 (5.9%) rare fusion genes, and 6 (17.6%) *ETV6ex4/NTRK3*. No mutations in codons 12, 13, and 61 of *K-*, *N-* and *H-RAS*, *BRAF^K601E^*, or *ETV6ex5/NTRK3* or *AKAP9/BRAF* were detected. Fusion genes were significantly more frequent than *BRAF^V600E^* (*p* = 0.002). Clinicopathologically, *RET/PTC3* was associated with solid growth pattern and higher tumor aggressiveness, *BRAF^V600E^* and *RET/PTC1* with classic papillary morphology and mild clinical phenotype, and *ETV6ex4/NTRK3* with follicular-patterned PTC and reduced aggressiveness. The spectrum of driver mutations in sporadic childhood PTC in Belarus largely parallels that in Chernobyl PTC, yet the frequencies of some oncogenes may likely differ from those in the early-onset Chernobyl PTC; clinicopathological features correlate with the oncogene type.

## 1. Introduction

**Figure 1:**
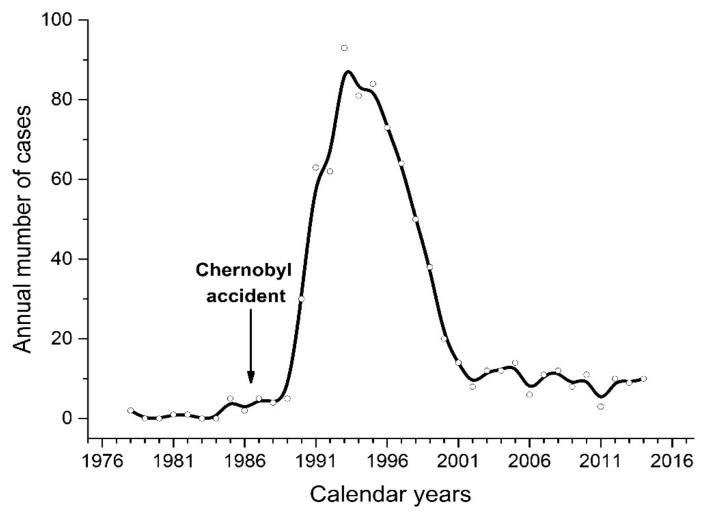
Number of cases of thyroid cancer in children aged 0–14 years in Belarus during 1978–2014. Data are retrieved from the database of Childhood Cancer SubRegistry of Belarus. Approximate population of children 0–14 years old was 1.57 million in 1986.

Thirty-five years have passed since the Chernobyl accident, which caused a sharp increase in the incidence of childhood thyroid cancer in the exposed population. According to the database of Childhood Cancer SubRegistry of Belarus, the peak of 3.79 cases per 100,000 in children aged up to 14 years inclusively at diagnosis was observed in 1995 (Figure 1). The vast majority of tumors were papillary thyroid carcinomas (PTCs) genetically characterized by a very high prevalence of gene rearrangements [1,2,3,4,5,6] and low frequency of point mutations [7,8,9,10,11].

Since 2001, only sporadic thyroid cancer occurs in children as all patients of corresponding age were born after January 1987 and were not exposed to Chernobyl radioiodines. During 2001–2012 in Belarus, age-standardized incidence rate for boys aged 0–14 years at diagnosis was 0.39, 0.86 for girls, and 0.62 per 100,000 for both genders. For comparison, in Italy in patients aged 0–14 years, age-standardized incidence rate of thyroid cancer was 0.20 and 0.39 for boys and girls, respectively, in 2003–2008 [12]. In the USA, during 1984–2010, age-standardized incidence rate of differentiated thyroid carcinoma was 0.05 for boys, 0.18 for girls, and 0.11 for both genders [13]. 

While no country-wide ultrasound screening of thyroid diseases is conducted in Belarus in children, thyroid cancer incidence is several-fold higher than those in western countries. In part, this could be explained by parental awareness of the consequences of Chernobyl motivating them to request thyroid ultrasound examination for their children even in the absence of symptoms or indications. The study of cancer incidence in children born to the A-bomb survivors in Hiroshima and Nagasaki has not found a link to parental radiation exposure doses [14]. The genetic study found no evidence of elevated rate of germline de novo mutations (that theoretically might contribute to transgenerational health effects) in children of parents exposed following the Chernobyl accident in Ukraine [15]. Therefore, the increased incidence of thyroid cancer in Belarus in non-exposed children is unlikely due to radiation factor or possible exposures, if any, of their parents.

A recent study presented clinical and morphological features of 127 sporadic PTCs from Belarus in children and adolescents (4–18 years old at surgery) born from 1987 to 1992 and operated between 1991 and 2010 [16], but no reports on genetic alterations in these tumors are available.

Here, we investigated the prevalence of common oncogenes (the most frequent fusion genes, and the *BRAF* and *RAS* point mutations) in 34 sporadic pediatric PTCs from Belarus in children (4–14 years old at surgery), and correlated molecular findings with clinicopathological characteristics. Comparisons of the obtained data with those from the literature were performed.

## 2. Results

### 2.1. Prevalence of Known Oncogenes in Childhood Sporadic PTC

A total of 34 patients operated on for PTC participated in this study. Mean age of patients at the time of operation was 12.4 ± 2.4 y.o., range 4–14 y.o; sex distribution: 12/34 (35.3%) boys and 22/34 (64.7%) girls; tumor size was from 6mm to 45 mm, mean 16.1 ± 9.0 mm. Nucleic acids extracted from paired normal and cancerous tissues were analyzed for the presence of *RET/PTC1*, *RET/PTC3*, *ETV6/NTRK3,* and *AKAP9/BRAF* gene rearrangements, and the *BRAF* and *RAS*-family point mutations. Overall, 61.8% (21/34) tumors harbored one of these oncogenes.

**Figure 2:**
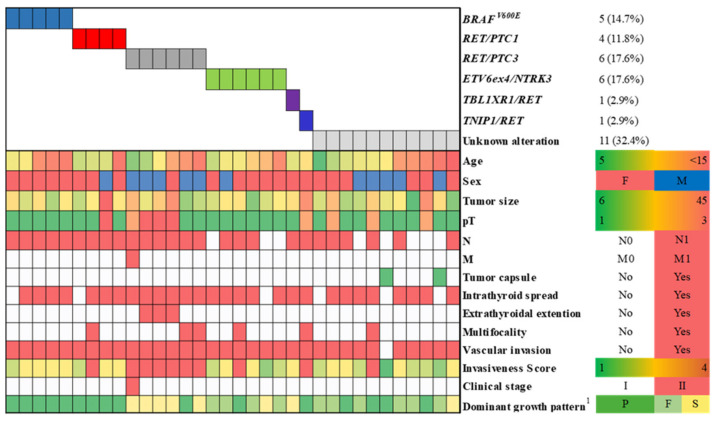
Distribution of driver oncogenes by clinicopathological characteristics of 34 childhood PTCs. ^1^ The dominant growth pattern: P—papillary, F—follicular, S—solid-trabecular.

The *BRAF^V600E^* mutation was detected in 5/34 (14.7%) of tumors (Figure 2, Supplementary Table S1). Rearranged *RET* occurred in 12/34 (35.3%) of tumors, including 4/34 *RET/PTC1* (11.8% or 4/12, 33.3% among *RET/PTC*-positive tissues), 6/34 *RET/PTC3* (17.6% or 6/12, 50.0% among all *RET/PTC* fusions) and two rare fusion genes from the *RET/PTC* family (2/34, 5.9%; the description of *TBL1XR1/RET* and *TNIP1/RET* is provided in Section 2.3 below); and 6/34 (17.6%) of tumors harbored *ETV6ex4/NTRK3*. The overall prevalence of childhood PTCs harboring these fusion genes was 18/34 (52.9%). *RET/PTC* rearrangements were more frequent (about two-fold) than *ETV6ex4/NTRK3* (*p* = 0.005). The prevalence of tumors with fusion genes was significantly higher than that with *BRAF^V600E^* (OR = 6.53, 95% CI 2.04–20.90, *p* = 0.002, adjusted for age and sex). All mutations were mutually exclusive. No mutations in codons 12, 13, and 61 of *K-*, *N-* and *H-RAS*, *BRAF^K601E^* or *ETV6ex5/NTRK3* or *AKAP9/BRAF* rearrangement were detected.

### 2.2. Association of Oncogenes with Clinicopathological Features

Statistical details of the descriptive univariate analysis of tumors with different genetic alterations are presented in Supplementary Table S2. On multivariate analysis (Table 1), tumors harboring *BRAFV600E* or *RET/PTC1* correlated with dominant papillary growth pattern; the correlation was statistically significant for *BRAF^V600E^* (OR = 6.49 (1.05–infinity), *p* = 0.046) and suggestive for *RET/PTC1* (15.09 (0.51–450.29), *p* = 0.078). *RET/PTC3* was associated with male sex (OR = 9.52 (1.17–76.92), *p* = 0.035), N1b category (OR = 9.54 (1.40–infinity), *p* = 0.025), the highest invasiveness score (OR = 4.41 (1.35–inf), *p* = 0.021), and solid-trabecular growth pattern (OR = 2.82 (1.03–inf), *p* = 0.045). *ETV6/NTRK3* was associated with follicular tumor architecture only (OR = 10.15 (1.26–81.81), *p* = 0.011). All *RET/PTC* rearrangements combined were associated with lateral lymph node involvement (OR = 8.92 (1.56–inf), *p* = 0.017), the highest invasiveness score (OR = 12.35 (1.93–78.96), *p* = 0.008), lower frequency of follicular growth pattern (OR = 0.02 (0.01–0.65), *p* = 0.027) and higher frequency of solid-trabecular growth pattern (OR = 6.67 (1.15–38.65), *p* = 0.034). Tumors with unknown driver mutation did not display statistically significant associations; they only weakly tended to be less likely accompanied by nodal disease, less likely to have high invasiveness score, and were likely to have follicular structure.

It should be noted that some statistically significant associations seen on univariate analysis could not be detected on multivariate analysis due to insufficient statistical power. For the same reason some estimates were unstable (e.g., those with the infinite limits of confidence intervals) yet for most instances provided meaningful result.

**Figure 3:**
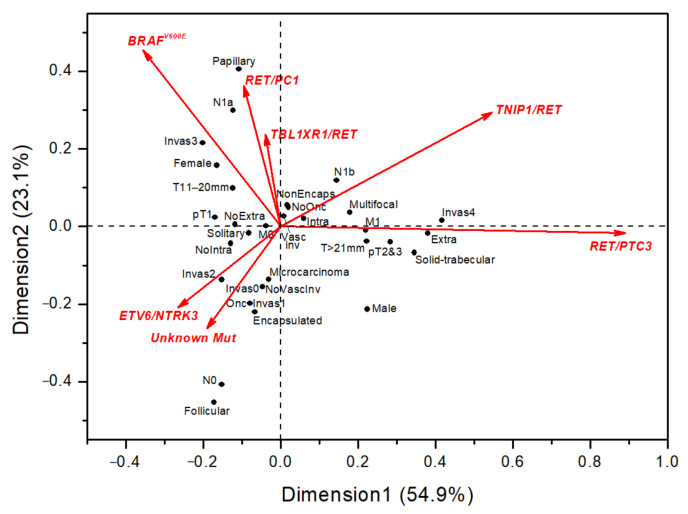
Correspondence analysis of clinicopathological characteristics (black circles) of childhood PTCs with respect to oncogenic drivers (red arrows). The smaller angle between the types of driver mutation indicates their stronger correlation (e.g., between *RET/PTC1* and *BRAF^V600E^*), while right or obtuse angles indicate a lack of correlation (note the position of *RET/PTC3* against other mutations). The smaller angle between clinicopathological variables indicates similarity in response pattern (e.g., an invasiveness score of 4 would be expected to frequently coexist with tumors displaying extrathyroidal extension). The distance between the positions of driver oncogenes and clinicopathological variables reflects, to some extent, the association between them (e.g., PTCs with *RET/PTC3* are likely to display solid-trabecular, while those with *RET/PTC1* display a papillary growth pattern more frequently). Dimension 1 and 2 accounted for 82.1% of variance cumulatively, and the dimensions 3–6 for the remaining 17.9% (not shown).

We also used correspondence analysis to graphically present clinicopathological associations of different oncogenes or unknown drivers (Figure 3). As follows from the results, except for *RET/PTC3*, none of the oncogenes or unknown drivers were associated with aggressive tumor features. *RET/PTC1* and *BRAF^V600E^* were rather associated with papillary tumor structure, but no associations with aggressive tumor behavior were evident. Of interest, tumors with unknown drivers were relatively close in their properties to those harboring *ETV6/NTRK3*. These tumors tended to have a follicular growth pattern, less frequent nodal disease, and less frequent aggressive features. Overall, the findings of the correspondence analysis were in line with the results of the regression analysis.

### 2.3. Detection of Novel Fusion Oncogenes by RNA-seq

**Figure 4:**
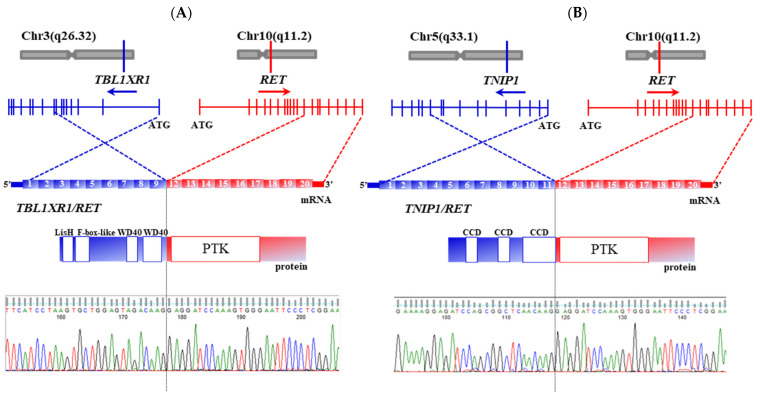
Characterization of the *TBL1XR1/RET* and *TNIP1/RET* fusions. (**A**) Genomic location, mRNA and protein structure, and validation by Sanger sequencing of *TBL1XR1/RET*. Exons 1–9 of *TBL1XR1* are shown in blue and exons 12–20 of *RET* are shown in red for mRNA. For protein: LisH (lissencephaly type-1-like homology) domain, a dimerization motif; F-box-like, mediates protein–protein interactions; WD40, mediates protein–protein interactions; PTK, protein tyrosine kinase. (**B**) Genomic location, mRNA and protein structure, and validation by Sanger sequencing of *TNIP1/RET*. Exons 1–11 of *TNIP1/RET* are shown in blue and exons 12–20 of *RET* are shown in red for mRNA. For protein: CCD—coiled coil domain, mediates homodimerization; PTK, protein tyrosine kinase. Sequences of *TBL1XR1/RET* and *TNIP1/RET* are presented in Supplementary File S1 and are deposited to GenBank under accession numbers MZ269488 and MZ269489, respectively.

The RNA-seq was performed for two tumors without known genetic alterations. One tumor displayed a fusion whose 5′-end contained a portion of *TBL1XR1* (transducin (beta)-like 1 X-linked receptor 1; located on chromosome 3q26.32), and the 3′-end—a portion of *RET* (located on chromosome 10q11.2) (Figure 4A). *TBL1XR1/RET* is a result of an interchromosomal translocation t(3;10) (q26.32;q11.2) that juxtaposes exons 1–9 of *TBL1XR1* to exons 12–20 of *RET*. The TBL1XR1 fragment includes LisH (lis homology domain) domain, which was reported to enable dimerization [17]. The breakpoint in *RET*, at the mRNA level, localized at the same place as in *RET/PTC1* and *RET/PTC3,* in exon 12. The tumor sized 17 mm displayed solid-follicular growth pattern, lymph node metastasis, intraglandular spread, vascular invasion and was confined to the thyroid, clinical stage I (Supplementary Table S1).

Besides the present work, *TBL1XR1/RET* was previously found in one patient from The Cancer Genome Atlas (TCGA) thyroid cancer series [18]. We therefore performed the functional study and found that *TBL1XR1/RET* transforms NIH 3T3 cells (Supplementary File S1, Supplementary Figure S1), can form homodimers (Supplementary Figure S2) and preferentially activates the PI3K/Akt pathway compared with moderate activation of the MAP-kinase cascade (Supplementary Figure S3). These findings demonstrate oncogenic properties of the *TBL1XR1/RET* fusion.

One more tumor harbored another previously undescribed *RET* fusion, *TNIP1/RET*. The 5′-end of this fusion is a portion of *TNIP1* (TNFAIP3 interacting protein 1; located on chromosome 5q13.2), and the 3′-end—a portion of *RET*. *TNIP1/RET* is a result of an interchromosomal translocation t(5;10) (q13.2; q11.2) juxtaposing exons 1–12 of *TNIP1* to exons 12–20 of *RET* (Figure 4B). The *TNIP1* fragment contains the coiled-coil domain enabling dimerization. The conventional breakpoint in *RET*, at the mRNA level, was in exon 12. The tumor sized 35 mm had papillary architecture, lymph node metastasis, intraglandular spread, vascular invasion and did not demonstrate extrathyroidal extension, stage I (Supplementary Table S1).

## 3. Discussion

This study is the first analysis of common driver mutations in children with sporadic PTC from Belarus. Before this work, a number of groups had investigated the mutational profile of PTC in children exposed to Chernobyl radiation in Ukraine and Belarus, countries located in the same geographic region and sharing a substantial part of environmental factors and dietary habits. With time, reports from different countries on molecular characteristics of PTCs in non-exposed young patients and in small series of PTCs in patients exposed to radiation, typically external beam therapy, are accumulating. Here, we perform an analysis of mutational frequencies from our study against the findings of other researches in different groups of young patients with PTC of different etiology.

### 3.1. BRAF^V600E^ Mutation in Radiation-Related and Sporadic Pediatric/Young Adult PTC Patients

#### 3.1.1. BRAF^V600E^ Mutation in Radiation-Related and Sporadic PTC from Belarus and Ukraine

**Figure 5:**
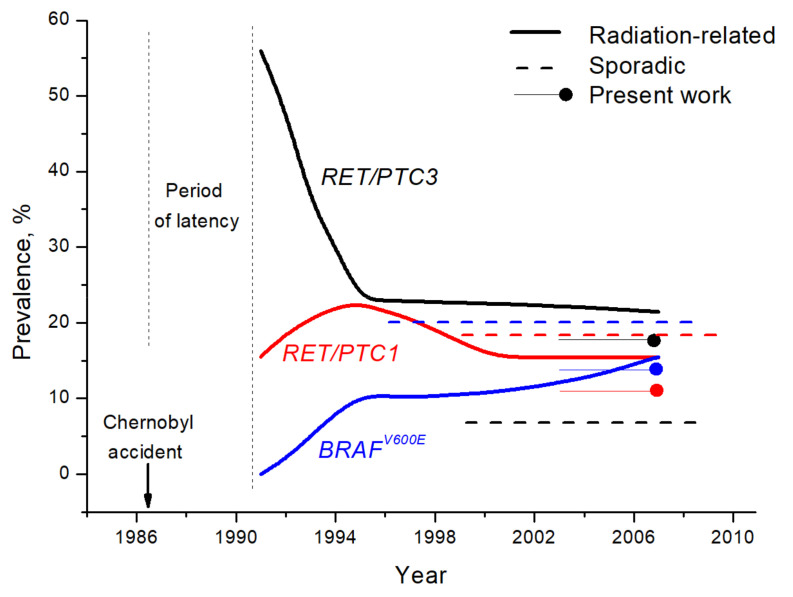
Tentative prevalence of *BRAF^V600E^* (blue lines), *RET/PTC1* (red lines), and *RET/PTC3* (black lines) in radiation-related and sporadic childhood/pediatric/young PTC patients from Belarus and Ukraine from 1991 to 2009. Data are summarized from the current work and the sources are listed in Supplementary Tables S2 and S3. The length of lines tentatively corresponds to the periods of sampling.

According to the available reports, the null mutant *BRAF* prevalence in early-onset (1991–1992) Chernobyl PTC rose to approximately 10% in mid-1990s, remained relatively stable at this level until the beginning of the 2000s, and then grew to about 15% until 2007 (Figure 5 and Supplementary Table S3). In sporadic PTC, *BRAF^V600E^* was absent among eight non-exposed children from Ukraine aged ≤ 15 years [10]. The 31.2% (10/32) prevalence was found in a recent study of non-exposed PTC patients from Ukraine aged less than 18 years in Ukraine [19], and a similar prevalence of 25.9% (7/27) was reported in children and adolescents aged 5–19 years in an independent study [20]. For comparison, in a group of adult patients with sporadic PTC aged 47 ± 9.7 years from Russia, *BRAF^V600E^* was found in 61.8% (47/76) of cases [21].

**Figure 6:**
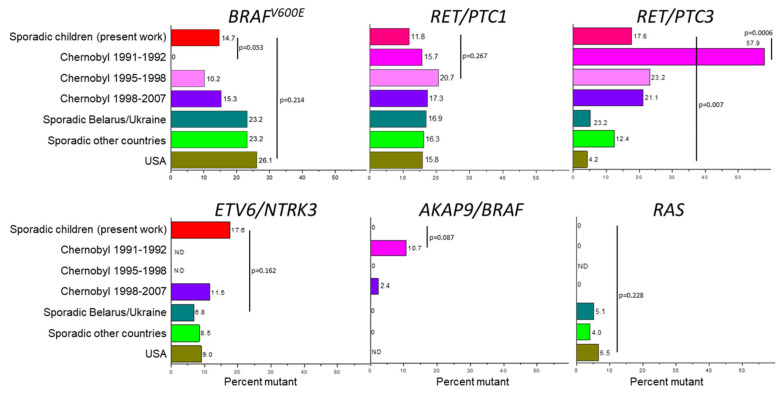
Prevalence (%) of the major driver oncogenes analyzed in this work in childhood sporadic PTC from Belarus and those in other relevant groups of young patients with radiation-related (Chernobyl) and sporadic PTC. Percent mutant is presented on the same scale enabling direct visual comparison between different types of oncogenes. Each bar is labeled with corresponding prevalence. ND—not determined due to the lack of data. The most relevant/meaningful statistical comparisons are shown (all other comparisons returned statistically insignificant results).

Although the small sample sizes both in the present work and in relevant studies (see Supplementary Table S3) do not provide the desired statistical power, our results suggest that the prevalence of *BRAF^V600E^* in sporadic childhood PTC from Belarus (this work, 5/34, 14.7%) may likely be somewhat higher than in early-onset (1991–1992) pediatric Chernobyl PTC (0%, 0/34, *p* = 0.053, Figure 6—*BRAF^V600E^*). It is slightly higher, but statistically insignificant, than that in radiation-related childhood PTC from Belarus and Ukraine diagnosed in 1995–2001 (10.2%, 13/127 on pooled analysis, *p* = 0.540) and comparable to that in 1998–2007 (15.3%, 21/137, *p* = 1.0). No significant difference was found from sporadic pediatric cases from Ukraine diagnosed during 1999–2009 (23.2%, 23/99, *p* = 0.340). The *BRAF^V600E^* frequency in our childhood series, however, is significantly lower (*p* = 4 × 10^−5^) than in sporadic adult PTC in patients from Russia [21].

#### 3.1.2. BRAF^V600E^ Mutation in Radiation-Related and Sporadic PTC from Different Countries

The prevalence of *BRAF^V600E^* in radiation-related pediatric/young patient PTC from different countries other than Belarus and Ukraine is 0% (0/16, although patient groups are small, Table S3), which is not statistically significant from that in our series (*p* = 0.163). The prevalence of *BRAF^V600E^* in sporadic PTCs ranged from 0% to 56%, pooled 23.2% (283/1218), and again, was not statistically significant from that in our series (*p* = 0.305).

Several recent investigations in the U.S. have challenged the notion of very low prevalence of *BRAF^V600E^* in pediatric PTC [22,23,24,25,26,27,28,29]. The prevalence of *BRAF^V600E^* in all studies from the U.S. is 26.1% (99/380, pooled), which is not statistically significant from that in our series (*p* = 0.214). Although being noticeably higher than in the first reports on childhood PTC studies, this relatively high prevalence of *BRAF^V600E^* remains significantly lower than that in TCGA PTC series [18], 58.5% (235/402, patients aged 15–89 years, median 46.8 years old, principally adults not exposed to radiation, *p* = 4 × 10^−20^). This additionally attests to the difference in the *BRAF^V600E^* prevalence between pediatric and adult PTC. 

It is worth noting that despite the difference in *BRAF^V600E^* prevalence between our series and that (pooled) in pediatric sporadic PTC from other countries is formally non-significant, there is a large variation in the prevalence of *BRAF^V600E^* between the individual studies. This suggests that whenever the comparisons of mutation prevalence/frequency between different studies are being addressed, it may be necessary to also consider, in addition to the age distribution, the country of origin and, perhaps, patients’ ethnicity. Data from the same geographic areas could likely be interpreted more adequately.

### 3.2. RET/PTC1 and RET/PTC3 Rearrangements in Pediatric/Young Adult PTC Patients

Our analysis of the literature on *RET/PTC* rearrangements in pediatric Chernobyl PTC showed distinct patterns of *RET/PTC1* and *RET/PTC3* prevalence in tumors with different periods of latency (Figure 5, Supplementary Table S4). The prevalence of *RET/PTC1* grew slightly from 16% in the earliest Chernobyl PTCs to ~21% in mid-1990s and then returned to ~17% in the latest pediatric tumors operated at the end of 1990s–2000s. *RET/PTC1* prevalence of 11.8% (4/34) in our sporadic childhood series does not differ—either meaningfully or statistically significantly—from those in radiation-related PTC for any of these three periods of time (*p* = 0.740, 0.267 and 0.603, respectively, Figure 5-*RET/PTC1*). 

In contrast, the proportion of radiation-related tumors harboring *RET/PTC3* was the highest, ~58%, in the beginning of the 1990s, then declined to 23% in the mid-1990s and remained at approximately this level or was slightly declining until the first decade of the 2000s (Figure 4). The prevalence of *RET/PTC3* of 17.6% (6/34) in our series differs significantly only from that in the earliest exposed cases (57.9%, 22/38, *p* = 0.0006, Figure 5-*RET/PTC3*) but not from the later-onset radiation-related tumors (mid-1990s: 23.2%, 91/392, *p* = 0.530, and later-onset: 21.1%, 28/133, *p* = 0.813). Nevertheless, these findings emphasize the association of this type of gene rearrangement with the younger age of PTC patients and radiation exposure reported in a recent meta-analysis [30].

Non-exposed pediatric groups of PTC patients from Ukraine had 16.9% (10/59) and 5.1% (3/59) of cases with *RET/PTC1* and *RET/PTC3*, respectively [18,19]. This prevalence does not differ statistically significantly from those in our sporadic childhood series for either oncogene (*p* = 0.563 and 0.069, respectively).

The prevalence of *RET/PTC1* and *RET/PTC3* rearrangements in sporadic pediatric PTC from different countries varies from 0 to 100% with extremal numbers pertaining to small series (Supplementary Table S4, Figure 6—*RET/PTC1* and *RET/PTC3*). The pooled prevalence is 16.3% (97/595) for *RET/PTC1* and 12.4% (74/595) for *RET/PTC3* for the countries other than Ukraine. Comparison of our childhood series with pooled data for all countries indicate a non-significant difference for both *RET/PTC1* and *RET/PTC3* (*p* = 0.634 and 0.423, respectively). Comparison with pooled data from the U.S., however, shows the difference for *RET/PTC3* (4.2%, 11/265, *p* = 0.007) but not for *RET/PTC1* (15.8%, 42/265, *p* = 0.800). This suggests that comparisons of the prevalence of *RET/PTC* rearrangements in PTCs from different regions would be recommended for adjustment for geographical origin of tumors and patients’ age.

### 3.3. ETV6/NTRK3 Rearrangement in Pediatric/Young Adult PTC Patients

*ETV6/NTRK3* in pediatric PTC was determined previously in a few works (Supplementary Table S5, Figure 6—*ETV6/NTRK3*). In patients from Ukraine exposed to Chernobyl radiation, this fusion gene was found in 11.5% (12/104), and in 6.8% (4/59) of non-exposed cases [18,19]. Young-exposed PTC patients from the UkrAm cohort (mean age 22.7 years) displayed the prevalence of 14.5% (9/62) [31].

In the reports from other countries, a 9.0% (12/133) prevalence was found cumulatively in four studies of sporadic PTCs from the U.S. [29,32,33,34]. An 8.6% (3/35) prevalence was reported in a Brazilian study [35], and an 11.1% (1/9) in childhood PTC from Japan [36]. The pooled prevalence in all these studies was 8.5% (24/284). Statistical comparisons with our childhood group (17.6%, 6/34) did not reveal significant difference (*p* > 0.3 in any test). At variance with other activated oncogenes, the prevalence of *ETV6/NTRK3* does not appear to display pronounced geographic heterogeneity, apparently ranging from 3% to 20% in PTCs from young patients.

### 3.4. AKAP9/BRAF Rearrangement in Pediatric/Young Adult PTC Patients

We did not find *AKAP9/BRAF* in our series in contrast to the short-latency (5–6 years after exposure, Figure 6—*AKAP9/BRAF*) Chernobyl PTC from Belarussian children aged at operation 11.4 ± 3.6 years, in whom 10.7% (3/28) of tumors harbored this type of rearrangement [37] (Supplementary Table S5). In the same work, however, no *AKAP9/BRAF* rearrangements were found among 64 PTCs diagnosed after the 9–12 years long period of latency. The prevalence of 0% (0/16) [19] and 3.8% (1/26) [20] was reported in radiation-related pediatric PTCs from Ukraine. These two studies also did not find *AKAP9/BRAF* in 32 and 27 sporadic pediatric PTCs from Ukraine, respectively. In our previous analysis of young PTC patients from Fukushima, no *AKAP9/BRAF* was found among 9 children aged 9–14 years [36]. Where possible, statistical comparisons of *AKAP9/BRAF* prevalence indicated nonsignificant difference with our PTC group (the strongest *p* = 0.087). Together, these data indicate that *AKAP9/BRAF* is a relatively rare event even in exposed pediatric PTC patients; this fusion gene is virtually absent in sporadic childhood PTC.

### 3.5. The RAS Family Gene Mutations in PTC Diagnosed in Pediatric/Young Adult Patients

Mutations in the *RAS* family genes were absent in our childhood group, similarly to previous reports on early-onset pediatric radiation-related PTC from Belarus [38,39]. Three studies of radiation-related pediatric Ukrainian PTCs diagnosed at the end of the 1990s and during the first decade of the 2000s in Ukraine also reported the null prevalence of *RAS* mutations [8,17,18] (0/15, 0/26 and 0/16, respectively, Supplementary Table S5, Figure 6—*RAS*). The two latter studies also reported the 7.4% (2/27) and 3.1% (1/32), respectively, prevalence of *NRAS* codon 61 mutations in young patients from Ukraine not exposed to radiation [18,19].

In sporadic pediatric PTC series from other countries, the prevalence of *RAS* point mutations ranged from 0 to 25% [8,9,24,28,36,40,41,42,43,44], pooled 4.0% (20/498) without significant overall difference with our series (*p* = 0.631), demonstrating again that this type of oncogene is rather rare in PTC in young patients.

### 3.6. Correlation between the Mutational Status and Clinicopathological Characteristics

Our analysis of the relationships between driver oncogene and clinicopathological characteristics in PTC was generally in line with previous findings in radiation-related [3,4,5,6,18] and sporadic pediatric/young patient thyroid cancers [3,20,32,40,43,45]. 

Tumors with *BRAF^V600E^* or *RET/PTC1* are known to associate with classic papillary morphology. In our series, all PTCs harboring *BRAF^V600E^* or *RET/PTC1* displayed a dominant papillary growth pattern. This correlation was confirmed on univariate statistical analysis for both oncogenes; on multivariate analysis the statistical significance was preserved for *BRAF^V600E^* and remained suggestive for *RET/PTC1*. *RET/PTC1* and *BRAF^V600E^* did not confer tumor aggressiveness, in agreement with previous reports on the lack of overly aggressive features in *BRAF^V600E^*-positive PTCs in pediatric patients [23,36,40,41,43,46].

In contrast, tumors driven by *RET/PTC3* displayed the most aggressive phenotype. In our series, *RET/PTC3* was associated with the highest invasiveness score and solid-trabecular growth pattern. Compared with *RET/PTC1*, *RET/PTC3* has been shown to display greater mitogenic potential when transfected to the cells, and yielded higher phosphorylation levels of ERK1/2 [47]. Transgenic murine models also demonstrated greater oncogenicity of *RET/PTC3* than *RET/PTC1* [48,49,50]. In clinical settings, Chernobyl-related childhood PTC from Belarus with *RET/PTC3* was associated with the shorter period of latency and more advanced tumor stage [3,4,6]. As follows from literature data, the frequency of *RET/PTC3* declined in the Chernobyl PTC over time (Figure 5 and Figure 6, Supplementary Table S4), as did tumor aggressiveness as described in our recent analysis [51]. The more pronounced aggressiveness of tumors with *RET/PTC3* was also reported in PTC patients from China [52]. Note that in our series, the only tumor with distant metastasis to the lung in a 12-year-old boy harbored *RET/PTC3*. Most probably, the correlations with tumor aggressiveness for all tumors harboring any type of *RET/PTC* (see Table S1) were also due to *RET/PTC3*. All these observations are in support of the notion that *RET/PTC3* may confer a more aggressive clinical phenotype.

The *ETV6ex4/NTRK3* rearrangement in our series was strongly associated with the follicular tumor architecture, which is also concordant with previous reports [20,27,29]. Although one study of pediatric PTC in northeast United States suggested the aggressiveness of *ETV6ex4/NTRK3*-positive tumors [29], our work demonstrates the lack of such in childhood PTC from Belarus. Despite the prevalence of *ETV6ex4/NTRK3,* it does not seem to display marked geographical difference; tumor characteristics may apparently vary. In our opinion, this may indicate that tumor phenotype may be modified by complex gene–environmental interactions such as dietary factors, for example.

It is also interesting to note that PTCs with unknown driver oncogene, which accounted for about one-third of all cases in our study, were relatively close to those harboring *ETV6ex4/NTRK3* on correspondence analysis (Figure 3). They were more likely to display follicular growth pattern and rather less aggressive clinical phenotype. A limitation of our study, along with a relatively small sample size, is that we did not analyze other known fusion genes, such as *PAX8-PPARG* and *AGK-BRAF*, which may be expected to occur in follicular-patterned PTCs with an appreciable frequency [53,54,55].

## 4. Materials and Methods

### 4.1. Patients and Tissue Samples

Snap-frozen tumor (T) and normal (N) thyroid tissue counterparts from 34 pathologically confirmed PTCs from patients born 2–16 years after the Chernobyl accident who were operated in Minsk City Oncological Dispensary during 2001–2007 were available for the study. Informed consent was obtained from the parents/guardians of each patient from the childhood group and from each adult patient. The study protocol was approved by the Ethical Committees of Minsk City Oncological Dispensary and Nagasaki University.

### 4.2. Nucleic acid Extraction

Normal or tumor thyroid tissues were homogenized in ISOGEN reagent (Nippon Gene, Toyama, Japan), and both RNA and DNA were then extracted from the same homogenized specimen. Total RNA was isolated according to the manufacturer’s protocol. DNA was extracted from the interphase and organic phase using DNA extraction buffer (4M guanidine thiocyanate, 50 mM sodium citrate, 1M Tris base, no pH adjustment) followed by isopropanol precipitation.

### 4.3. Detection of Known Fusion Oncogenes and Point Mutations

Genomic DNA was used to determine mutational status of *BRAF* (exon15), and *H-, K-, N-RAS* (codons 12, 13, 61) by direct DNA sequencing. For PCR amplification, we used AmpliTaq Gold Polymerase (Applied Biosystems, Austin, TX, USA). PCR product was treated with ExoSAP-IT (GE Healthcare, Cleveland, OH, USA) and sequenced using Big Dye Terminator sequencing kit version 3.1 (Applied Biosystems, Austin, TX, USA) on an ABI3730 automated sequencer (Applied Biosystems, Foster City, CA, USA). Total RNA from N and T tissues was reverse transcribed using SuperScript III RT (Invitrogen, Vilnius, Lithuania). Fragments of *RET/PTC1*, *RET/PTC3*, *AKAP9/BRAF,* and *ETV6/NTRK3* fusion genes were PCR-amplified using primers encompassing corresponding breakpoints in cDNA. PCR products were electrophoresed in agarose gels, transferred to nylon membrane (Roche, Penzberg, Germany), and hybridized with digoxigenin-11-ddUTP (Roche, Mannheim, Germany) labeled oligonucleotide probe spanning the junction of each rearrangement. Primer and probe sequences are listed in Supplementary Table S6.

### 4.4. RNA-seq and Data Analysis

Total RNA from two tumors with unknown driver oncogene was treated with DNase I and purified by RNeasy Micro Kit (Qiagen, Germantown, MD, USA). Library preparation and Illumina HiSeq2000 sequencing were performed at Takara Bio facility (Takara Bio Inc., Shiga, Japan). Sequence read analysis for gene fusion was performed using deFuse and Circos software packages. Characterization of novel fusion genes and relevant analyses are presented in Supplementary File S1.

### 4.5. Statistical Analysis

In addition to conventional clinicopathological characteristics, we introduced a synthetic variable termed “invasiveness score”. The invasiveness score is the arithmetic sum of every instance of N1, M1, intrathyroid spread, extrathyroidal extension, multifocality or lymphatic/vascular invasion (encoded 0 for the absence and 1 for the presence of a given feature in a given tumor), either isolated or in combination with other(s), for each tumor [56]. Thus defined, the invasiveness score ranged from 0 to 4; the latter was the maximum observed in this childhood PTC series.

Fishers’ exact test, Freeman–Halton test, Cochran–Armitage test or Cramer’s V effect size measure were used for categorical data; nonparametric tests were applied to continuous variables. Logistic regression analyses with very small numbers of outcomes (<5 per cell) or when quasi-complete separation was observed were conducted using Firth’s approach to bias-reducing penalized maximum likelihood fit or exact logistic regression. Statistical assessments were performed using SAS 9.4 version of SAS (SAS Institute Inc., Cary, NC, USA) or IBM SPSS Statistics 24 software (International Business Machines Corp., Armonk, NY, USA). All *p*-values were 2-sided and considered significant if *p* < 0.05.

Correspondence analysis was performed using R with packages ca, FactoMineR, factoextra, gplots, tidyverse, and corrplot installed. The chosen graphic output of correspondence analysis was a biplot displaying columns (here, the driver oncogenes) in principal coordinates and rows (categorical clinicopathological variables) in contribution coordinates.

## 5. Conclusions

Our investigation detected common oncogenic drivers in about two-thirds of PTCs diagnosed in non-exposed to radiation children from Belarus. The overall frequencies of oncogenes were the following: *RET/PTC3* = *ETV6ex4/NTRK3 > BRAF^V600E^* > *RET/PTC1*. Fusion genes occurred significantly more frequently than point mutations, which is typical for childhood PTC. Among fusion oncogenes, *RET/PTC* species were the most common, with *RET/PTC3* being the most frequent. The lower frequency of *RET/PTC3* in sporadic PTC compared with the early-onset (5–6 years period of latency) Chernobyl PTC was the only difference between the two etiological forms of childhood PTC. In contrast, *BRAF^V600E^* likely occurred more frequently in sporadic PTC than in Chernobyl PTC developing after the short period of latency.

Clinicopathological correlations of different oncogenes paralleled those reported in radiation-related PTC. *RET/PTC3* was associated with the highest number of aggressive features while *BRAF^V600E^* and *RET/PTC1* conferred milder tumor phenotype, and tumors with *ETV6ex4/NTRK3* or an unknown oncogene were the least aggressive. 

Thus, the spectrum of driver mutations in sporadic childhood PTC largely overlaps with that in radiation-related PTC, and clinicopathological features correlate with the oncogene regardless of tumor etiology.

## Figures and Tables

**Table 1:** Association of mutational status with clinicopathological features in sporadic childhood PTC ^1^.

		*BRAF^V600E^*	*RET/PTC1*	*RET/PTC3*	*ETV6ex4/NTRK3*	*TBL1XR1/RET*	*TNIP1/RET*	Any *RET/PTC*	Unknown
		5 (14.7%)	4 (11.8%)	6 (17.6%)	6 (17.6%)	1 (2.9%)	1 (2.9%)	12 (35.3%)	11 (32.4%)
Sex (ref = F)	OR (95% CI)	0.13 (0.01–3.33) ^2^		**9.52 (1.17–76.92) ^2^**					
	*p*-value	0.215		**0.035**					
Tumor size ≥ 21 mm	OR (95% CI)						9.00 (0.47–inf)	2.88 (0.53–15.61)	
	*p*-value						0.100	0.219	
pT category 2+3 *vs.* 1	OR (95% CI)			3.86 (0.43–34.44)			6.00 (0.32–inf)	3.89 (0.70–21.67)	0.85 (0.15–4.81)
	*p*-value			0.227			0.143	0.122	0.849
Lymph node metastasis	OR (95% CI)							19.78 (0.92–425.24)	0.22 (0.04–1.20)
	*p*-value							0.057	0.080
N1a	OR (95% CI)	7.85 (0.49–126.31)							
	*p*-value	0.146							
N1b	OR (95% CI)			**9.54 (1.40–inf)**				**8.92 (1.56–inf)**	
	*p*-value			**0.025**				**0.017**	
Distant metastasis	OR (95% CI)			7.16 (0.28–184.32)					
	*p*-value			0.235					
Multifocality	OR (95% CI)						1.00 (0.05–inf)		
	*p*-value						0.500		
Extrathyroidal extension	OR (95% CI)			18.73 (0.73–483.93)				7.07 (0.40–123.84)	
	*p*-value			0.077				0.181	
Tumor capsule	OR (95% CI)					3.90 (inf–inf)			8.34 (0.50–138.58)
	*p*-value					0.998			0.139
Invasiveness score									
3	OR (95% CI)			0.15 (0.01–3.80)					
	*p*-value			0.251					
4	OR (95% CI)			**4.41 (1.35–inf)**				**12.35 (1.93–78.96)**	0.15 (0.02–1.27)
	*p*-value			**0.021**				**0.008**	0.081
Dominant component									
papillary	OR (95% CI)	**6.49 (1.05–inf ^3^)**	15.09 (0.51–450.29)						
	*p*-value	**0.046**	0.078						
follicular	OR (95% CI)				**10.15 (1.26–81.81)**			**0.02 (0.01–0.65)**	4.54 (0.93–22.31)
	*p*-value				**0.011**			**0.027**	0.062
solid-trabecular	OR (95% CI)			**2.82 (1.03–inf)**		3.40 (0.18–inf)		**6.67 (1.15–38.65)**	
	*p*-value			**0.045**		0.227		**0.034**	

^1^ Only those associations that displayed signs of statistical significance on univariate analysis (Supplementary Table S2) are shown; all other tests returned non-significant results and are not shown. ORs are adjusted for age at operation and sex unless otherwise specified. ^2^ adjusted for age at operation; ^3^ infinity. Numbers in bold indicate statistically significant associations.

## Data Availability

Datasets on oncogene prevalence in PTCs from children or young patients generated during this study are publicly available from the literature and are presented in supplementary material. The RNA-seq datasets generated and analyzed during the current study may be made available from the corresponding author on reasonable request, subject to approval by the Ethics Committees of Minsk City Clinical Oncologic Dispensary and Nagasaki University. Sequences of the *TBL1XR1/RET* and *TNIP1/RET* fusion genes derived from the RNA-seq data analysis are presented in Supplementary File S1 and are submitted to GenBank under accession numbers MZ269488 and MZ269489, respectively.

## Supplementary Materials

The following are available online at https://www.mdpi.com/article/10.3390/cancers13133374/s1, Supplementary File S1: Functional analysis of the TBL1XR1-RET and description of the TNIP1/RET fusion oncogenes, Table S1: Clinicopathological features of sporadic childhood PTCs according to the oncogenes, Table S2: Statistical assessment of clinicopathological features of sporadic childhood PTCs according to the oncogene, Table S3: Frequency of the *BRAF^V600E^* mutation in pediatric PTC of radiation and sporadic etiology in different countries, Table S4: Frequency and prevalence of *RET/PTC1* and *RET/PTC3* rearrangements in pediatric PTC of radiation and sporadic etiology in different countries, Table S5: Frequency and prevalence of *ETV6/NTRK3* and *AKAP9/BRAF* rearrangements, and of point mutations in the *RAS* family genes in pediatric PTC of radiation and sporadic etiology in different countries, Table S6: Primer and probe sequences for detections of oncogenes. References [57,58,59,60,61,62,63,64,65,66,67,68,69,70,71,72,73,74,75,76,77,78,79,80,81,82,83,84,85,86,87] are referred to in Supplementary Materials.
