# Supplementary material for: Major Oncogenic Drivers and Their Clinicopathological Correlations in Sporadic Childhood Papillary Thyroid Carcinoma in Belarus
Source: Cancers (Basel). 2021 Jul 5;13(13):3374. doi: 10.3390/cancers13133374 (PMC8268670; doi:10.3390/cancers13133374)
Supplement: Supplementary file 1 [file cancers-13-03374-s001.zip › cancers-1264129-supplementary.pdf]

**Table S1.** Clinicopathological features of sporadic childhood PTCs according to the oncogenes

| | *BRAF*$^{V600E}$ | *RET/PTC1* | *RET/PTC3* | *ETV6ex4/NTRK3* | *TBL1XR1/RET*[1] | *TNIP1/RET*[1] | Any *RET/PTC* | Unknown |
|---|---|---|---|---|---|---|---|---|
| Number of patients | 5 (14.7%) | 4 (11.8%) | 6 (17.6%) | 6 (17.6%) | 1 (2.9%) | 1 (2.9%) | 12 (35.3%) | 11 (32.4%) |
| Age, mean ± SD, years | 13.6 ± 1.2 | 11.7 ± 2.0 | 11.9 ± 3.2 | 12.6 ± 1.5 | 11.4 | 13.0 | 11.9 ± 2.4 | 12.2 ± 3.1 |
| Sex, F/M (%F) | 5/0 (100%) | 3/1 (75%) | 1/5 (16.7%) | 5/1 (83.3%) | 1/0 (100%) | 1/0 (100%) | 6/6 (50%) | 6/5 (54.5%) |
| Tumors size, mean ± SD, mm | 14.4 ± 3.4 | 21.3 ± 15.9 | 19.3 ± 10.1 | 12.8 ± 2 .9 | 17 | 35 | 21.1 ± 11.7 | 15.7 ± 8.5 |
| microcarcinoma | 1 (20%) | 0 | 1 (16.7%) | 1 (16.7%) | 0 | 0 | 1 (8.3%) | 3 (27.3%) |
| 11-20 mm | 4 (80%) | 3 (75%) | 2 (33.3%) | 5 (83.3%) | 1 (100%) | 0 | 6 (50.0%) | 5 (45.5%) |
| ≥ 21 mm | 0 | 1 (25%) | 3 (50.0%) | 0 | 0 | 1 (100%) | 5 (41.7%) | 3 (27.3%) |
| pT category | | | | | | | | |
| 1 | 5 (100%) | 3 (75%) | 2 (33.3%) | 6 (100%) | 1 (100%) | 0 | 6 (50%) | 8 (72.7%) |
| 2 | 0 | 0 | 1 (16.7%) | 0 | 0 | 1 (100%) | 2 (16.7%) | 3 (27.3%) |
| 3 | 0 | 1 (25%) | 3 (50.0%) | 0 | 0 | 0 | 4 (33.3%) | 0 |
| Lymph node metastasis | 5 (100%) | 4 (100%) | 6 (100%) | 3 (50%) | 1 (100%) | 1 (100%) | 12 (100%) | 6 (54.5%) |
| N1a | 2 (40%) | 1 (25%) | 0 | 0 | 0 | 0 | 1 (8.3%) | 1 (9.1%) |
| N1b | 3 (60%) | 3 (75%) | 6 (100%) | 3 (50%) | 1 (100%) | 1 (100%) | 11 (91.7%) | 5 (45.5%) |
| Distant metastasis | 0 | 0 | 1 (16.7%) | 0 | 0 | 0 | 1 (8.3%) | 0 |
| Multifocality | 0 | 1 (25%) | 2 (33.3%) | 1 (16.7%) | 0 | 1 (100%) | 4 (33.3%) | 1 (9.1%) |
| Intrathyroidal spread | 4 (80%) | 3 (75%) | 6 (100%) | 5 (83.3%) | 1 (100%) | 1 (100%) | 11 (91.7%) | 8 (72.7%) |
| Extrathyroidal extension | 0 | 0 | 3 (50.0%) | 0 | 0 | 0 | 3 (25.0%) | 0 |
| Vascular invasion | 5 (100%) | 4 (100%) | 6 (100%) | 6 (100%) | 1 (100%) | 1 (100%) | 12 (100%) | 10 (90.9%) |
| Tumor capsule | 0 | 0 | 0 | 0 | 0 | 0 | 0 | 2 (18.2%) |
| Invasiveness score | | | | | | | | |
| 0 | 0 | 0 | 0 | 0 | 0 | 0 | 0 | 1 (9.1%) |
| 1 | 0 | 0 | 0 | 1 (16.7%) | 0 | 0 | 0 | 1 (9.1%) |
| 2 | 1 (20%) | 1 (25%) | 0 | 2 (33.3%) | 0 | 0 | 1 (8.3%) | 4 (36.4%) |
| 3 | 4 (80%) | 2 (50%) | 0 | 2 (33.3%) | 1 (100%) | 0 | 3 (25.0%) | 4 (36.4%) |
| 4 (max) | 0 | 1 (25%) | 6 (100%) | 1 (16.7%) | 0 | 1 (100%) | 8 (66.7%) | 1 (9.1%) |
| Clinical stage | | | | | | | | |
| I | 5 (100%) | 4 (100%) | 5 (83.3%) | 6 (100%) | 1 (100%) | 1 (100%) | 11 (91.7%) | 1 (100%) |
| II | 0 | 0 | 1 (16.7%) | 0 | 0 | 0 | 1 (8.3%) | 0 |
| Dominant component | | | | | | | | |
| papillary | 5 (100%) | 4 (100%) | 1 (16.7%) | 1 (16.7%) | 0 | 1 (100%) | 6 (50.0%) | 4 (36.4%) |
| follicular | 0 | 0 | 0 | 4 (66.7%) | 0 | 0 | 0 | 6 (54.5%) |
| solid-trabecular | 0 | 0 | 5 (83.3%) | 1 (16.7%) | 1 (100%) | 0 | 6 (50.0%) | 1 (9.1%) |

[1] these fusion genes were discovered using RNA-seq as described in details in the corresponding subsection below

**Table S2.** Statistical assessment of clinicopathological features of sporadic childhood PTCs according to the oncogene[1]

| | ***BRAF^V600E*** | ***RET/PTC1*** | ***RET/PTC3*** | ***ETV6ex4/NTRK3*** | ***TBL1XR1/RET*** | ***TNIP1/RET*** | **Any *RET/PTC*** | **Unknown** |
|---|---|---|---|---|---|---|---|---|
| Number of patients (%) | 5 (14.7%) | 4 (11.8%) | 6 (17.6%) | 6 (17.6%) | 1 (2.9%) | 1 (2.9%) | 12 (35.3%) | 11 (32.4%) |
| Age at operation, years | 0.196; 0.193 | 0.364; -0.131 | 0.775; -0.046 | 0.912; -0.020 | 0.588; -0.110 | 0.941; 0.022 | 0.292; -0.156 | 0.856; 0.029 |
| Sex | 0.137; **0.307** | 1.0; 0.079 | **0.014**; **0.465** | 0.389; 0.180 | 1.0; 0.129 | 1.0; 0.129 | 0.265; 0.227 | 0.459; 0.147 |
| Tumor size, mm | 0.962; -0.011 | 0.630; 0.071 | 0.612; 0.077 | 0.364; 0.136 | 0.647; 0.090 | 0.176; 0.218 | 0.146; 0.218 | 0.490; -0.103 |
| microcarcinoma | 1.0; 0.026 | 1.0; 0.169 | 1.0; 0.012 | 1.0; 0.012 | 1.0; 0.081 | 1.0; 0.081 | 0.389; 0.180 | 0.363; 0.175 |
| 11-20 mm | 0.379; 0.179 | 0.627; 0.120 | 0.202; 0.240 | 0.364; 0.231 | 1.0; 0.146 | 0.412; 0.208 | 0.487; 0.132 | 0.458; 0.188 |
| ≥ 21 mm | 0.309; 0.230 | 1.0; 0.013 | 0.126; 0.289 | 0.297; 0.257 | 1.0; 0.097 | 0.235; **0.314** | 0.098; **0.316** | 1.0; 0.061 |
| pT category | 0.348; 0.249 | 0.511; 0.199 | **0.005**; **0.560** | 0.269; 0.278 | 0.831; 0.104 | **0.050**; **0.419** | **0.012**; **0.508** | 0.160; **0.328** |
| pT1 | 0.293; 0.249 | 1.0; 0.012 | **0.031**; **0.422** | 0.162; 0.278 | 1.0; 0.104 | 0.265; 0.290 | **0.040**; **0.394** | 1.0; 0.013 |
| pT2 and pT3 | 0.293; 0.249 | 1.0; 0.012 | **0.031**; **0.422** | 0.162; 0.278 | 1.0; 0.104 | 0.265; 0.290 | **0.040**; **0.394** | 1.0; 0.013 |
| Lymph node metastasis | 0.309; 0.230 | 0.551; 0.203 | 0.297; 0.257 | 0.126; 0.289 | 1.0; 0.097 | 1.0; 0.097 | **0.030**; **0.410** | 0.079; **0.357** |
| N1a | 0.094; **0.364** | 0.409; 0.150 | 1.0; 0.169 | 1.0; 0.169 | 1.0; 0.064 | 1.0; 0.064 | 1.0; 0.079 | 1.0; 0.057 |
| N1b | 1.0; 0.041 | 1.0; 0.079 | 0.069; **0.342** | 0.641; 0.142 | 1.0; 0.129 | 1.0; 0.129 | **0.024**; **0.417** | 0.138; 0.279 |
| Distant metastasis (M1) | 1.0; 0.072 | 1.0; 0.064 | 0.176; **0.376** | 1.0; 0.081 | 1.0; 0.030 | 1.0; 0.030 | 0.353; 0.236 | 1.0; 0.120 |
| Multifocality | 0.559; 0.192 | 0.559; 0.070 | 0.281; 0.190 | 1.0; 0.012 | 1.0; 0.081 | 0.176; **0.376** | 0.154; **0.304** | 0.638; 0.155 |
| Intrathyroidal spread | 1.0; 0.026 | 0.559; 0.070 | 0.562; 0.214 | 1.0; 0.012 | 1.0; 0.081 | 1.0; 0.081 | 0.389; 0.180 | 0.363; 0.175 |
| Extrathyroidal extension | 1.0; 0.129 | 1.0; 0.114 | **0.003**; **0.672** | 1.0; 0.144 | 1.0; 0.054 | 1.0; 0.054 | **0.037**; **0.421** | 0.535; 0.215 |
| Vascular invasion | 1.0; 0.072 | 1.0; 0.064 | 1.0; 0.081 | 1.0; 0.081 | 1.0; 0.030 | 1.0; 0.030 | 1.0; 0.129 | 0.324; 0.252 |
| Tumor capsule | 1.0; 0.104 | 1.0; 0.091 | 1.0; 0.116 | 1.0; 0.116 | 1.0; **0.595** | 1.0; 0.044 | 0.529; 0.185 | 0.098; **0.361** |
| Invasiveness score | 0.322; **0.381** | 1.0; 0.133 | **0.001**; **0.717** | 0.617; 0.266 | 1.0; 0.221 | 0.618; 0.270 | **0.008**; **0.620** | 0.167; **0.413** |
| 0 | 1.0; 0.072 | 1.0; 0.064 | 1.0; 0.081 | 1.0; 0.081 | 1.0; 0.030 | 1.0; 0.030 | 1.0; 0.129 | 0.324; 0.252 |
| 1 | 1.0; 0.104 | 1.0; 0.091 | 1.0; 0.116 | 0.326; 0.212 | 1.0; 0.044 | 1.0; 0.044 | 0.529; 0.185 | 1.0; 0.094 |
| 2 | 1.0; 0.035 | 1.0; 0.013 | 0.297; 0.257 | 0.609; 0.107 | 1.0; 0.097 | 1.0; 0.097 | 0.210; 0.265 | 0.388; 0.209 |
| 3 | 0.059; **0.357** | 0.627; 0.088 | 0.062; **0.364** | 1.0; 0.047 | 0.382; 0.221 | 1.0; 0.137 | 0.292; 0.201 | 1.0; 0.027 |
| 4 | 0.291; 0.268 | 1.0; 0.035 | **<0.001**; **0.717** | 0.644; 0.129 | 1.0; 0.112 | 0.294; 0.270 | **0.001**; **0.604** | 0.113; **0.308** |
| Dominant component | 0.063; **0.440** | 0.125; **0.387** | **0.001**; **0.656** | 0.077; **0.384** | 0.235; **0.314** | 1.0; 0.185 | **0.004**; **0.569** | 0.087; **0.396** |
| papillary | **0.016**; **0.440** | **0.039**; **0.387** | 0.180; 0.282 | 0.180; 0.282 | 1.0; 0.164 | 0.471; 0.185 | 1.0; 0.044 | 0.477; 0.148 |
| follicular | 0.291; 0.268 | 0.296; 0.236 | 0.148; 0.081 | **0.048**; **0.378** | 1.0; 0.112 | 1.0; 0.112 | **0.006**; **0.477** | **0.045**; **0.381** |
| solid-trabecular | 0.309; 0.230 | 0.551; 0.203 | **0.001**; **0.653** | 1.0; 0.075 | 0.235; **0.314** | 1.0; 0.097 | **0.013**; **0.461** | 0.227; 0.235 |
| Oncocytic changes | 1.0; 0.104 | 1.0; 0.091 | 1.0; 0.116 | 0.326; 0.212 | 1.0; 0.044 | 1.0; 0.044 | 0.529; 0.185 | 1.0; 0.094 |

[1] each cell contains two numbers: first is a p-value from the Mann-Whitney (age and tumor size) or Fisher exact test (all other categorial variables); the second is Kendall's tau-b rank correlation coefficient (age and tumor size) or Cramer's V effect size (all other categorial variables); the magnitude of the effect size: V~0.1 small, V~0.3 medium, V~0.5 large

**Table S3.** Frequency of the *$BRAF^{V600E}$* mutation in pediatric PTC of radiation and sporadic etiology in different countries

| Country | Patients' age | N | *$BRAF^{V600E}$* | % | Sampling | Reference[1] |
|---|---|---|---|---|---|---|
| | | | *Radiation-related* | | | |
| Belarus | 6-20 | 34 | 0 | 0 | 1991-1992 | [1] |
| Belarus | 16.0 ± 5.0 | 64 | 10 | 15.6 | 1995-1998 | [2] |
| Belarus/Ukraine | 12-31 | 21 | 2 | 9.5 | 1995-1998 | [1] |
| Ukraine | 13.8 (10.3-15.7) | 27 | 1 | 3.5 | 1995-2001 | [3] |
| Ukraine | 14.3 | 15 | 0 | 0 | 1998-2000 | [4] |
| Ukraine | 17.7 (14-24) | 33 | 8 | 24.2 | NS (2016)[1,2] | [5] |
| Ukraine | 18.0 (13 - 23) | 26 | 2 | 7.7 | 1998-2007 | [6] |
| Ukraine | 27.1 | 62 | 9 | 14.5 | 1998-2007 | [7] |
| Ukraine | 15.9 (13-18) | 16 | 2 | 12.5 | 1998-2004 | [8] |
| Czech Republic | NS (6-20) | 2 | 0 | 0 | 2003-2017 | [9] |
| Canada | <18 | 2 | 0 | 0 | NS (2018) | [10] |
| Colombia | 11.8 | 1 | 0 | 0 | 2009-2018 | [11] |
| Brazil | ≤18 | 3 | 0 | 0 | NS (2019) | [12] |
| USA | <10-23 | 8 | 0 | 0 | 2005-2016 | [13] |
| | | | *Sporadic* | | | |
| Belarus | 12.4 (2-16) | 34 | 5 | 14.7 | 2001-2007 | Present study |
| Ukraine | 11.9 (7.9-15.1) | 8 | 0 | 0 | 1995-2001 | [3] |
| Ukraine | 15.6 (5-19) | 27 | 7 | 25.9 | 1999-2009 | [6] |
| Ukraine | 16.3 (7-21) | 32 | 6 | 18.8 | NS (2016)[2,3] | [5] |
| Ukraine | 15.4 (10-18) | 32 | 10 | 31.3 | 1998-2004 | [8] |
| Czech Republic | 16.3 (11-19) | 81 | 15 | 18.5 | 2003-2017 | [9] |
| Serbia | 8-18 | 8 | 1 | 12.5 | 1995-2008 | [14] |
| France/Italy | 8-19 | 28 | 3 | 10.7 | 2002-2010 | [15] |
| Portugal/UK | ≤18 | 17 | 1 | 5.9 | NS, (1963-1992 UK) | [16] |
| Portugal | 5-17 | 11 | 0 | 0 | 2002-2007 | [17] |
| Turkey | 16 (6-18) | 50 | 15 | 30 | 1995-2015 | [18] |
| Turkey | 12.4 (1-17) | 56 | 14 | 25 | 1983-2015 | [19] |
| Saudi Arabia | ≤18 | 84 | 9 | 10.7 | 1998-2015 | [20] |
| Saudi Arabia | 12-18 | 3 | 1 | 33.3 | 1987-2006 | [21] |
| Saudi Arabia | 17 (5-18) | 48 | 9 | 18.8 | 2004-2019 | [22] |
| India | ≤20 | 98 | 14 | 14.3 | 2005-2018 | [23] |
| China | 3-13 | 48 | 17 | 35.4 | 1994-2014 | [24] |
| China | <21 | 30 | 16 | 53.3 | 2015-2018 | [25] |
| Japan | ≤20 | 81 | 44 | 54.3 | 1991-2013 | [26] |
| Japan | 11.3 (< 15) | 31 | 1 | 3.2 | 1962-1995 | [4] |
| Japan | 12.7 (9-14) | 9 | 3 | 33.3 | 2013-2014 | [27] |

| | | | | | | |
|---|---|---|---|---|---|---|
| Brazil | ≤18 | 35 | 3 | 8.6 | NS (2017) | [28] |
| Brazil | ≤18 | 77 | 12 | 15.6 | NS (2019) | [12] |
| Colombia | 11.8 | 15 | 1 | 6.3 | 2009-2018 | [11] |
| USA | 17.5 (10-21) | 20 | 0 | 0 | NS (2005) | [29] |
| USA | 15 (10-17) | 20 | 4 | 20 | 1985-2004 | [30] |
| USA | ≤21 | 11 | 2 | 18.2 | 2007-2011 | [31] |
| USA | 12-16 | 5 | 2 | 40 | 2009-2012 | [32] |
| USA | 10-19 | 27 | 10 | 37 | 2009-2014 | [33] |
| USA | 14 (12-17) | 13 | 4 | 30.8 | 2008-2012 | [34] |
| USA | 13.7 (2-18) | 19 | 7 | 36.8 | 1999-2012 | [35] |
| USA | 14.7 (7.9-18.4) | 34 | 9 | 26.5 | 2001-2013 | [36] |
| USA | 0-15 | 9 | 5 | 55.6 | 1973-2005 | [37] |
| USA | 15 (4-18) | 62 | 12 | 19.4 | 1989-2012 | [38] |
| USA | 12-22 | 8 | 2 | 25 | NS (2017) | [39] |
| USA | <17 | 8 | 0 | 0 | NS (2017) | [40] |
| USA | 0-21 | 50 | 24 | 48.0 | 2003-2015 | [41] |
| USA | <10-23 | 31 | 6 | 19.4 | 2005-2016 | [13] |
| USA | 14 (6-18) | 63 | 12 | 19.0 | 1989-2012 | [42] |
| Canada | 14.4 (10-17) | 28 | 5 | 17.9 | NS (2018) | [10] |

[1] NS, not specified (year of publication); [2] estimated period 1999-2009

**Table S4.** Frequency and prevalence of *RET/PTC1* and *RET/PTC3* rearrangements in pediatric PTC of radiation and sporadic etiology in different countries

| Country | Patients' age | N | *RET/PTC1* (%) | *RET/PTC3* (%) | Sampling | Reference |
|---|---|---|---|---|---|---|
| | | | *Radiation-related* | | | |
| Belarus | 8-19 | 12 | 2 (16.7) | 6 (50) | 1993-1995 | [43] |
| Belarus | 15-18 | 38 | 6 (15.8) | 22 (57.9) | 1991-1992 | [44] |
| Belarus | 8-15 | 51 | 12 (23.5) | 12 (23.5) | 1993-1997 | [45] |
| Belarus, Ukraine | < 15 | 61 | 13 (21.3) | 3 (4.9) | 1990-1994 | [46] |
| Belarus, Ukraine | < 15 | 45 | 7 (15.6) | 12 (26.7) | 1995-1996 | [46] |
| Belarus | 12.9 (11-17) | 32 | 9 (28.1) | 1 (3.1) | 1996 | [47,48] |
| Belarus | 6-18 | 39 | 9 (23.1) | 11 (28.8) | 1995-1997 | [49] |
| Ukraine | 6-18 | 28 | 5 (17.9) | 12 (42.9) | 1995-1997 | [49] |
| Belarus | ≤14 | 99 | 19 (19.2) | 26 (26.3) | 1993-1998 | [50] |
| Belarus | 12.0 (10-17) | 25 | 5 (20.0) | 8 (32.0) | NS (2001)[1] | [51] |
| Ukraine | 12-31 | 21 | 2 (9.5) | 6 (28.6) | 1995-1998 | [1] |
| Ukraine | 11-18 | 8 | 1 (12.5) | 3 (37.5) | NS (2005) | [52] |
| Ukraine | 18.0 (13-23) | 26 | 4 (15.4) | 7 (26.9) | 1998-2007 | [6] |
| Ukraine | 20.1-23.6 (20-29) | 62 | 14 (22.6) | 8 (12.9) | 1998-2007 | [7] |
| Ukraine | 15.9 (13-18) | 16 | 2 (12.5) | 4 (25.0) | 1998-2004 | [8] |
| Czech Republic | NS (6-20) | 2 | 0 | 0 | 2003-2017 | [9] |
| Canada | <18 | 2 | 0 | 0 | NS (2018) | |
| | | | *Sporadic* | | | |
| Belarus | 12.4 (2-16) | 34 | 4 (11.8) | 6 (17.6) | 2001-2007 | Present study |
| Ukraine | 15.6 (5-19) | 27 | 5 (18.5) | 2 (7.4) | 1999-2009 | [6] |
| Ukraine | 15.4 (10-18) | 32 | 5 (15.6) | 1 (3.1%) | 1998-2004 | [8] |
| Czech Republic | NS (6-20) | 81 | 12 (14.8) | 6 (7.4) | 2003-2017 | [9] |
| Serbia | 8-18 | 8 | 5 (62.5) | 0 | 1992-2008 | [14] |
| France | 9-15 | 4 | 0 | 1 (25.0) | NS (1997) | [53] |
| Italy | 16 (8-18) | 25 | 5 (20) | 4 (16.0) | NS (2001) | [51] |
| Italy/France | 8-19 | 28 | 2 (7.1) | 6 (21.4) | 2002-2010 | [15] |
| Italy | 9-16 | 4 | 0 (0) | 1 (25.0) | NS (2002) | [54] |
| Italy | <19 | 9 | 3 (33.3) | 1 (11.1) | NS (1996) | [55] |
| Saudi Arabia | 12-18 | 3 | 0 | 0 | 1987-2006 | [21] |
| Saudi Arabia | 17 (5-18) | 48 | 9 (18.8) | 4 (8.3) | 2004-2019 | [22] |
| China | ≤20 | 6 | 3 (50) | | 2005-2006 | [56] |
| China/HK | 12 | 1 | 0 | 1 (100) | NS (2002) | [57] |
| Japan | 11-19 | 31 | 10 (32.3) | 4 (12.9) | NS (2005) | [58] |
| Japan | 12.8 (9-14) | 10 | 2 (20) | 1 (10.0) | 1980-1995 | [59] |
| Japan | 12.7 (9-14) | 9 | 3 (33.3) | 0 | 2013-2014 | [27] |

| | | | | | | |
|---|---|---|---|---|---|---|
| Brazil | ≤18 | 35 | 6 (17.1) | 6 (17.1) | NS (2017) | [28] |
| USA | 9 | 1 | 1 (100) | 0 | NS (2004) | [60] |
| USA | 18 (6-21) | 33 | 8 (24.2) | 2 (6.1) | NS (2002) | [61] |
| USA | 15-18 | 23 | 8 (34.8) | 3 (13.0) | since 1974 | [44] |
| USA | ≤21 | 11 | 3 (27.3) | 0 | 2007-2011 | [31] |
| USA | NS | 5 | 1 (20.0) | 0 | 2009-2012 | [32] |
| USA | 14 (12-17) | 13 | 1 (7.7) | 1 (7.7) | 2008-2012 | [34] |
| USA | 14.7 (7.9-18.4) | 34 | 5 (14.7) | 1 (2.9) | 2001-2013 | [36] |
| USA | 15 (4-18) | 62 | 10 (16.1) | 2 (3.2) | 1989-2012 | [38] |
| USA | 12-22 | 8 | 1 (12.5) | 0 | NS (2017) | [39] |
| USA | 17.5 (11-21) | 12 | 5 (41.7) | 2 (16.7) | NS (2005) | [29] |
| USA | 14 (6-18) | 63 | 5 (7.9) | 4 (6.3) | 1989-2012 | [42] |
| Canada | 14.0 (10-17) | 28 | 3 (10.7) | 4 (14.3) | NS (2018) | [10] |

[1] NS (2001), not specified (year of publication)

**Table S5.** Frequency and prevalence of *ETV6/NTRK3* and *AKAP9/BRAF* rearrangements, and of point mutations in the *RAS* family genes in pediatric PTC of radiation and sporadic etiology in different countries

| Country | Patients' age | N | Positive | % | Sampling | Reference |
|---|---|---|---|---|---|---|
| | | | ***ETV6/NTRK3*** | | | |
| | | | *Radiation-related* | | | |
| Ukraine | 18.0 (5-19) | 26 | 2 | 7.7 | 1998-2007 | [6] |
| Ukraine | 22.7 (14-32) | 62 | 9 | 14.5 | 1998-2008 | [62] |
| Ukraine | 15.9 (13-18) | 16 | 1 | 6.3 | 1998-2004 | [8] |
| | | | *Sporadic* | | | |
| Belarus | 12.4 (2-16) | 34 | 6 | 17.6 | 2001-2007 | Present study |
| Ukraine | 15.6 (5-19) | 27 | 2 | 7.4 | 1999-2009 | [6] |
| Ukraine | 15.4 (10-18) | 32 | 2 | 6.3 | 1998-2004 | [8] |
| Saudi Arabia | 17 (5-18) | 48 | 4 | 8.3 | 2004-2019 | [22] |
| Japan | 12.7 (9-14) | 9 | 1 | 11.9 | 2013-2014 | [27] |
| Brazil | ≤18 | 35 | 3 | 8.6 | NS (2017)[1] | [28] |
| USA | 13 (8-17) | 18 | 3 | 16.7 | 2002-2014 | [63] |
| USA | 15 (6-18) | 27 | 5 | 18.5 | 2009-2015 | [64] |
| USA | <10-23 | 25 | 2 | 8.0 | 2005-2016 | [13] |
| USA | 14 (6-18) | 63 | 2 | 3.2 | 1989-2012 | [42] |
| | | | ***AKAP9/BRAF*** | | | |
| | | | *Radiation-related* | | | |
| Belarus | 11.4 ± 3.6 | 28 | 3 | 10.7 | 1991-1992 | [2] |
| Belarus | 16.0 ± 5.0 | 64 | 0 | 0 | 1995-1998 | [2] |
| Ukraine | 18.0 (5-19) | 26 | 1 | 3.8 | 1998-2007 | [6] |
| Ukraine | 15.9 (13-18) | 16 | 0 | 0 | 1998-2004 | [8] |
| | | | *Sporadic* | | | |
| Belarus | 12.4 (2-16) | 34 | 0 | 0 | 2001-2007 | Present study |
| Ukraine | 15.6 (5-19) | 27 | 0 | 0 | 1999-2009 | [6] |
| Ukraine | 15.4 (10-18) | 32 | 0 | 0 | 1998-2004 | [8] |
| Japan | 12.7 (9-14) | 9 | 0 | 0 | 2013-2014 | [27] |
| | | | ***RAS*** | | | |
| | | | *Radiation-related* | | | |
| Belarus | 5-19 | 33 | 0 | 0 | 1991-1992 | [65] |
| Belarus | NS, "children" | 34 | 0 | 0 | 1992 | [66] |
| Ukraine | 14.3 | 15 | 0 | 0 | 1998-2000 | [4] |
| Ukraine | 18.0 (5-19) | 26 | 0 | 0 | 1998-2007 | [6] |
| Ukraine | 20-29 | 62 | 5 | 8.0 | 1998-2008 | [62] |

| | | | | | | |
|---|---|---|---|---|---|---|
| Ukraine | 15.9 (13-18) | 16 | 0 | 0 | 1998-2004 | [8] |
| Czech Republic | NS (6-20) | 2 | 1 | 50.0 | NS (2003-2017)[2] | [9] |
| | | | *Sporadic* | | | |
| Belarus | 12.4 (2-16) | 34 | 0 | 0 | 2001-2007 | Present study |
| Ukraine | 15.6 (5-19) | 27 | 2 | 7.4 | 1999-2009 | [6] |
| Ukraine | 15.4 (10-18) | 32 | 1 | 3.1 | 1998-2004 | [8] |
| Czech Republic | NS (6-20) | 81 | 1 | 1.2 | NS (2003-2017) | [9] |
| The Netherlands | 12.8 (9-16) | 8 | 0 | 0 | NS (2001) | [67] |
| Italy/France | 8-19 | 28 | 1 | 3.6 | 2002-2010 | [15] |
| Saudi Arabia | ≤18 | 79 | 2 | 2.5 | 1998-2015 | [20] |
| Saudi Arabia | 17 (5-18) | 48 | 2 | 4.2 | 2004-2019 | [22] |
| Japan | 11.3 | 31 | 0 | 0 | 1962-1995 | [4] |
| Japan | 12.7 (9-14) | 9 | 0 | 0 | 2013-2014 | [27] |
| USA | 17 (6-21) | 31 | 2 | 6.5 | NS (1999) | [68] |
| USA | 11-21 | 11 | 0 | 0 | NS (2005) | [29] |
| USA | 15 (4-18) | 62 | 4 | 6.5 | NS (2018) | [38] |
| USA | 12-22 | 8 | 2 | 25.0 | NS (2017) | [39] |
| USA | 14.7 (7-18) | 34 | 1 | 2.9 | 2001-2013 | [36] |
| USA | 12-16 | 5 | 1 | 20.0 | 2009-2012 | [32] |
| USA | 14 (6-18) | 63 | 4 | 6.4 | 1989-2012 | [42] |

[1] NS (2017), not specified (year of publication); [2] years of sampling in the study;

**Table S6**. Primer and probe sequences for detections of oncogenes

| Gene | Primer Sequence, 5'-3' | Annealing temperature (ºC) | Amplicon size (bp) | Reference | Southern blotting probe sequence, 5'-3' |
|---|---|---|---|---|---|
| *BRAF (ex15)* | ACATACTTATTGACTCTAAGAGGAAAGATGAA<br>GATTTTTGTGAATACTGGGAACTATGA | 60 | 401 | [27] | |
| *H-RAS (codons 12, 13)* | AGCAGGGCCCTCCTTGGCAG<br>CAGCCAGCCCTATCCTGGCTG | 65 | 261 | [27] | |
| *H-RAS (codon 61)* | CAGGGAGAGGCTGGCTGTGTG<br>CCACCTGTGCGGCGTGGGCT | 65 | 298 | [27] | |
| *K-RAS (codons 12, 13)* | GGTACTGGTGGAGTATTTGATAGT<br>CTCATGAAAATGGTCAGAGAAACCT | 60 | 290 | [27] | |
| *K-RAS (codon 61)* | GGTGCACTGTAATAATCCAGACTG<br>CTATAATTACTCCTTAATGTCAGCTT | 60 | 268 | [27] | |
| *N-RAS (codons 12 ,13)* | CACACTAGGGTTTTCATTTCCATTG<br>GGTAAAGATGATCCGACAAGTGAGCG | 63 | 283 | [27] | |
| *N-RAS (codon 61)* | TTGAACTTCCCTCCCTCCCTGC<br>AGCTCTATCTTCCCTAGTGTGGTAA | 65 | 316 | [27] | |
| *RET/PTC1* | GCCTGGAGGAGCTCACCAA<br>CTCTGCCTTTCAGATGGAA | 56 | 252 | [27] | GCGTTACCATCGAGGATCCAAA |
| *RET/PTC3* | ACCTGCCAGTGGTTATCAAGCT<br>TTCGCCTTCTCCTAGAGTTTTTCC | 59 | 154 | [27] | AGAACAGTCAGGAGGATCCAAA |
| *AKAP9/BRAF* | AGCAAGAACAGTTGATTTTGGA<br>GCAGACAAACCTGTGGTTGA | 56 | 181 | [2] | AAACTTCAGAAAGAACTCAATGTACTT |
| *ETV6(ex4)/NTRK3* | CATTCTTCCACCCTGGAAAC<br>TCCTCACCACTGATGACAGC | 55 | 97 | [62] | AACCATGAAGAAGGTCCCGT |
| *ETV6(ex5)/NTRK3* | AAGCCCATCAACCTCTCTCA<br>TCCTCACCACTGATGACAGC | 60 | 139 | [62] | AGAATAGCAGGTCCCGTGGC |
| *TBL1XR1/RET* | AAGATGTTCCAAGCAACAAGGATGTCACA<br>CCAAGTTCTTCCGAGGGAAT | 62 | 243 | Present study | AGTAGACAAGGAGGATCCAAA |
| *TNIP1/RET* | TCCAAGATTGAAATGGAGGAGACC<br>TTCGCCTTCTCCTAGAGTTTTTCC | 58 | 216 | Present study | GCTCAACAAGGAGGATCCAA |

**References**


1. Nikiforova, M.N.; Ciampi, R.; Salvatore, G.; Santoro, M.; Gandhi, M.; Knauf, J.A.; Thomas, G.A.; Jeremiah, S.; Bogdanova, T.I.; Tronko, M.D., et al. Low prevalence of BRAF mutations in radiation-induced thyroid tumors in contrast to sporadic papillary carcinomas. *Cancer Lett* **2004**, *209*, 1-6, doi:10.1016/j.canlet.2003.12.004.
2. Ciampi, R.; Knauf, J.A.; Kerler, R.; Gandhi, M.; Zhu, Z.; Nikiforova, M.N.; Rabes, H.M.; Fagin, J.A.; Nikiforov, Y.E. Oncogenic AKAP9-BRAF fusion is a novel mechanism of MAPK pathway activation in thyroid cancer. *J Clin Invest* **2005**, *115*, 94-101, doi:10.1172/JCI23237.
3. Powell, N.; Jeremiah, S.; Morishita, M.; Dudley, E.; Bethel, J.; Bogdanova, T.; Tronko, M.; Thomas, G. Frequency of BRAF T1796A mutation in papillary thyroid carcinoma relates to age of patient at diagnosis and not to radiation exposure. *J Pathol* **2005**, *205*, 558-564, doi:10.1002/path.1736.
4. Kumagai, A.; Namba, H.; Saenko, V.A.; Ashizawa, K.; Ohtsuru, A.; Ito, M.; Ishikawa, N.; Sugino, K.; Ito, K.; Jeremiah, S., et al. Low frequency of BRAFT1796A mutations in childhood thyroid carcinomas. *J Clin Endocrinol Metab* **2004**, *89*, 4280-4284, doi:10.1210/jc.2004-0172.
5. Handkiewicz-Junak, D.; Swierniak, M.; Rusinek, D.; Oczko-Wojciechowska, M.; Dom, G.; Maenhaut, C.; Unger, K.; Detours, V.; Bogdanova, T.; Thomas, G., et al. Gene signature of the post-Chernobyl papillary thyroid cancer. *Eur J Nucl Med Mol Imaging* **2016**, *43*, 1267-1277, doi:10.1007/s00259-015-3303-3.
6. Ricarte-Filho, J.C.; Li, S.; Garcia-Rendueles, M.E.; Montero-Conde, C.; Voza, F.; Knauf, J.A.; Heguy, A.; Viale, A.; Bogdanova, T.; Thomas, G.A., et al. Identification of kinase fusion oncogenes in post-Chernobyl radiation-induced thyroid cancers. *J Clin Invest* **2013**, *123*, 4935-4944, doi:10.1172/JCI69766.
7. Leeman-Neill, R.J.; Brenner, A.V.; Little, M.P.; Bogdanova, T.I.; Hatch, M.; Zurnadzy, L.Y.; Mabuchi, K.; Tronko, M.D.; Nikiforov, Y.E. RET/PTC and PAX8/PPARgamma chromosomal rearrangements in post-Chernobyl thyroid cancer and their association with iodine-131 radiation dose and other characteristics. *Cancer* **2013**, *119*, 1792-1799, doi:10.1002/cncr.27893.
8. Morton, L.M.; Karyadi, D.M.; Stewart, C.; Bogdanova, T.I.; Dawson, E.T.; Steinberg, M.K.; Dai, J.; Hartley, S.W.; Schonfeld, S.J.; Sampson, J.N., et al. Radiation-related genomic profile of papillary thyroid carcinoma after the Chernobyl accident. *Science* **2021**, *372*, doi:10.1126/science.abg2538.
9. Pekova, B.; Dvorakova, S.; Sykorova, V.; Vacinova, G.; Vaclavikova, E.; Moravcova, J.; Katra, R.; Vlcek, P.; Sykorova, P.; Kodetova, D., et al. Somatic genetic alterations in a large cohort of pediatric thyroid nodules. *Endocr Connect* **2019**, *8*, 796-805, doi:10.1530/EC-19-0069.
10. Wasserman, J.D.; Sabbaghian, N.; Fahiminiya, S.; Chami, R.; Mete, O.; Acker, M.;

Wu, M.K.; Shlien, A.; de Kock, L.; Foulkes, W.D. DICER1 Mutations Are Frequent in Adolescent-Onset Papillary Thyroid Carcinoma. *J Clin Endocrinol Metab* **2018**, *103*, 2009-2015, doi:10.1210/jc.2017-02698.

11. Castro, P.; Patino, E.; Fierro, F.; Rojas, C.; Buitrago, G.; Olaya, N. Clinical characteristics, surgical approach, BRAFV600E mutation and sodium iodine symporter expression in pediatric patients with thyroid carcinoma. *Journal of Pediatric Endocrinology and Metabolism* **2020**, DOI: 10.1515/jpem-2020-0201, in press, doi:10.1515/jpem-2020-0201.
12. Sisdelli, L.; Cordioli, M.; Vaisman, F.; Moraes, L.; Colozza-Gama, G.A.; Alves, P.A.G., Jr.; Araujo, M.L., Jr.; Alves, M.T.S.; Monte, O.; Longui, C.A., et al. AGK-BRAF is associated with distant metastasis and younger age in pediatric papillary thyroid carcinoma. *Pediatr Blood Cancer* **2019**, *66*, e27707, doi:10.1002/pbc.27707.
13. Potter, S.L.; Reuther, J.; Chandramohan, R.; Gandhi, I.; Hollingsworth, F.; Sayeed, H.; Voicu, H.; Kakkar, N.; Baksi, K.S.; Sarabia, S.F., et al. Integrated DNA and RNA sequencing reveals targetable alterations in metastatic pediatric papillary thyroid carcinoma. *Pediatr Blood Cancer* **2020**, 10.1002/pbc.28741, e28741, doi:10.1002/pbc.28741.
14. Stanojevic, B.; Dzodic, R.; Saenko, V.; Milovanovic, Z.; Pupic, G.; Zivkovic, O.; Markovic, I.; Djurisic, I.; Buta, M.; Dimitrijevic, B., et al. Mutational and clinico-pathological analysis of papillary thyroid carcinoma in Serbia. *Endocr J* **2011**, *58*, 381-393.
15. Sassolas, G.; Hafdi-Nejjari, Z.; Ferraro, A.; Decaussin-Petrucci, M.; Rousset, B.; Borson-Chazot, F.; Borbone, E.; Berger, N.; Fusco, A. Oncogenic alterations in papillary thyroid cancers of young patients. *Thyroid* **2012**, *22*, 17-26, doi:10.1089/thy.2011.0215.
16. Lima, J.; Trovisco, V.; Soares, P.; Maximo, V.; Magalhaes, J.; Salvatore, G.; Santoro, M.; Bogdanova, T.; Tronko, M.; Abrosimov, A., et al. BRAF mutations are not a major event in post-Chernobyl childhood thyroid carcinomas. *J Clin Endocrinol Metab* **2004**, *89*, 4267-4271, doi:10.1210/jc.2003-032224.
17. Espadinha, C.; Santos, J.R.; Sobrinho, L.G.; Bugalho, M.J. Expression of iodine metabolism genes in human thyroid tissues: evidence for age and BRAFV600E mutation dependency. *Clin Endocrinol (Oxf)* **2009**, *70*, 629-635, doi:10.1111/j.1365-2265.2008.03376.x.
18. Onder, S.; Ozturk Sari, S.; Yegen, G.; Sormaz, I.C.; Yilmaz, I.; Poyrazoglu, S.; Sanli, Y.; Giles Senyurek, Y.; Kapran, Y.; Mete, O. Classic Architecture with Multicentricity and Local Recurrence, and Absence of TERT Promoter Mutations are Correlates of BRAF (V600E) Harboring Pediatric Papillary Thyroid Carcinomas. *Endocr Pathol* **2016**, *27*, 153-161, doi:10.1007/s12022-016-9420-0.
19. Poyrazoglu, S.; Bundak, R.; Bas, F.; Yegen, G.; Sanli, Y.; Darendeliler, F.

Clinicopathological Characteristics of Papillary Thyroid Cancer in Children with Emphasis on Pubertal Status and Association with BRAF(V600E) Mutation. *J Clin Res Pediatr Endocrinol* **2017**, *9*, 185-193, doi:10.4274/jcrpe.3873.

20. Alzahrani, A.S.; Murugan, A.K.; Qasem, E.; Alswailem, M.; Al-Hindi, H.; Shi, Y. Single Point Mutations in Pediatric Differentiated Thyroid Cancer. *Thyroid* **2017**, *27*, 189-196, doi:10.1089/thy.2016.0339.
21. Zou, M.; Baitei, E.Y.; Alzahrani, A.S.; BinHumaid, F.S.; Alkhafaji, D.; Al-Rijjal, R.A.; Meyer, B.F.; Shi, Y. Concomitant RAS, RET/PTC, or BRAF mutations in advanced stage of papillary thyroid carcinoma. *Thyroid* **2014**, *24*, 1256-1266, doi:10.1089/thy.2013.0610.
22. Alzahrani, A.S.; Alswailem, M.; Alswailem, A.A.; Al-Hindi, H.; Goljan, E.; Alsudairy, N.; Abouelhoda, M. Genetic Alterations in Pediatric Thyroid Cancer Using a Comprehensive Childhood Cancer Gene Panel. *J Clin Endocrinol Metab* **2020**, *105*, doi:10.1210/clinem/dgaa389.
23. Chakraborty, D., Shakya, S., Ballal, S., Agarwal, S., Bal, C. BRAF V600E and TERT promoter mutations in paediatric and young adult papillary thyroid cancer and clinicopathological correlation. *Journal of Pediatric Endocrinology and Metabolism* **2020**, *in press*, doi:https://doi.org/10.1515/jpem-2020-0174.
24. Geng, J.; Wang, H.; Liu, Y.; Tai, J.; Jin, Y.; Zhang, J.; He, L.; Fu, L.; Qin, H.; Song, Y., et al. Correlation between BRAF (V600E) mutation and clinicopathological features in pediatric papillary thyroid carcinoma. *Sci China Life Sci* **2017**, *60*, 729-738, doi:10.1007/s11427-017-9083-8.
25. Huang, M.; Yan, C.; Wei, H.; Lv, Y.; Ling, R. Clinicopathological characteristics and prognosis of thyroid cancer in northwest China: A population-based retrospective study of 2490 patients. *Thorac Cancer* **2018**, *9*, 1453-1460, doi:10.1111/1759-7714.12858.
26. Oishi, N.; Kondo, T.; Nakazawa, T.; Mochizuki, K.; Inoue, T.; Kasai, K.; Tahara, I.; Yabuta, T.; Hirokawa, M.; Miyauchi, A., et al. Frequent BRAF (V600E) and Absence of TERT Promoter Mutations Characterize Sporadic Pediatric Papillary Thyroid Carcinomas in Japan. *Endocr Pathol* **2017**, *28*, 103-111, doi:10.1007/s12022-017-9470-y.
27. Mitsutake, N.; Fukushima, T.; Matsuse, M.; Rogounovitch, T.; Saenko, V.; Uchino, S.; Ito, M.; Suzuki, K.; Suzuki, S.; Yamashita, S. BRAF(V600E) mutation is highly prevalent in thyroid carcinomas in the young population in Fukushima: a different oncogenic profile from Chernobyl. *Sci Rep* **2015**, *5*, 16976, doi:10.1038/srep16976.
28. Cordioli, M.I.; Moraes, L.; Bastos, A.U.; Besson, P.; Alves, M.T.; Delcelo, R.; Monte, O.; Longui, C.; Cury, A.N.; Cerutti, J.M. Fusion Oncogenes Are the Main Genetic Events Found in Sporadic Papillary Thyroid Carcinomas from Children. *Thyroid* **2017**, *27*, 182-188, doi:10.1089/thy.2016.0387.

29. Penko, K.; Livezey, J.; Fenton, C.; Patel, A.; Nicholson, D.; Flora, M.; Oakley, K.; Tuttle, R.M.; Francis, G. BRAF mutations are uncommon in papillary thyroid cancer of young patients. *Thyroid* **2005**, *15*, 320-325, doi:10.1089/thy.2005.15.320.
30. Rosenbaum, E.; Hosler, G.; Zahurak, M.; Cohen, Y.; Sidransky, D.; Westra, W.H. Mutational activation of BRAF is not a major event in sporadic childhood papillary thyroid carcinoma. *Mod Pathol* **2005**, *18*, 898-902, doi:10.1038/modpathol.3800252.
31. Monaco, S.E.; Pantanowitz, L.; Khalbuss, W.E.; Benkovich, V.A.; Ozolek, J.; Nikiforova, M.N.; Simons, J.P.; Nikiforov, Y.E. Cytomorphological and molecular genetic findings in pediatric thyroid fine-needle aspiration. *Cancer Cytopathol* **2012**, *120*, 342-350, doi:10.1002/cncy.21199.
32. Buryk, M.A.; Monaco, S.E.; Witchel, S.F.; Mehta, D.K.; Gurtunca, N.; Nikiforov, Y.E.; Simons, J.P. Preoperative cytology with molecular analysis to help guide surgery for pediatric thyroid nodules. *Int J Pediatr Otorhinolaryngol* **2013**, *77*, 1697-1700, doi:10.1016/j.ijporl.2013.07.029.
33. Ballester, L.Y.; Sarabia, S.F.; Sayeed, H.; Patel, N.; Baalwa, J.; Athanassaki, I.; Hernandez, J.A.; Fang, E.; Quintanilla, N.M.; Roy, A., et al. Integrating Molecular Testing in the Diagnosis and Management of Children with Thyroid Lesions. *Pediatr Dev Pathol* **2016**, *19*, 94-100, doi:10.2350/15-05-1638-OA.1.
34. Gertz, R.J.; Nikiforov, Y.; Rehrauer, W.; McDaniel, L.; Lloyd, R.V. Mutation in BRAF and Other Members of the MAPK Pathway in Papillary Thyroid Carcinoma in the Pediatric Population. *Arch Pathol Lab Med* **2016**, *140*, 134-139, doi:10.5858/arpa.2014-0612-OA.
35. Givens, D.J.; Buchmann, L.O.; Agarwal, A.M.; Grimmer, J.F.; Hunt, J.P. BRAF V600E does not predict aggressive features of pediatric papillary thyroid carcinoma. *Laryngoscope* **2014**, *124*, E389-393, doi:10.1002/lary.24668.
36. Nikita, M.E.; Jiang, W.; Cheng, S.M.; Hantash, F.M.; McPhaul, M.J.; Newbury, R.O.; Phillips, S.A.; Reitz, R.E.; Waldman, F.M.; Newfield, R.S. Mutational Analysis in Pediatric Thyroid Cancer and Correlations with Age, Ethnicity, and Clinical Presentation. *Thyroid* **2016**, *26*, 227-234, doi:10.1089/thy.2015.0401.
37. Henke, L.E.; Perkins, S.M.; Pfeifer, J.D.; Ma, C.; Chen, Y.; DeWees, T.; Grigsby, P.W. BRAF V600E mutational status in pediatric thyroid cancer. *Pediatr Blood Cancer* **2014**, *61*, 1168-1172, doi:10.1002/pbc.24935.
38. Mostoufi-Moab, S.; Labourier, E.; Sullivan, L.; LiVolsi, V.; Li, Y.; Xiao, R.; Beaudenon-Huibregtse, S.; Kazahaya, K.; Adzick, N.S.; Baloch, Z., et al. Molecular Testing for Oncogenic Gene Alterations in Pediatric Thyroid Lesions. *Thyroid* **2018**, *28*, 60-67, doi:10.1089/thy.2017.0059.
39. Shifrin, A.L.; Fischer, M.; Paul, T.; Erler, B.; Gheysens, K.; Baodhankar, P.; Song-Yang, J.W.; Taylor, S.; Timmaraju, V.A.; Topilow, A., et al. Mutational analysis of metastatic lymph nodes from papillary thyroid carcinoma in adult and pediatric

patients. *Surgery* **2017**, *161*, 176-187, doi:10.1016/j.surg.2016.10.002.

40. Vanden Borre, P.; Schrock, A.B.; Anderson, P.M.; Morris, J.C., 3rd; Heilmann, A.M.; Holmes, O.; Wang, K.; Johnson, A.; Waguespack, S.G.; Ou, S.I., et al. Pediatric, Adolescent, and Young Adult Thyroid Carcinoma Harbors Frequent and Diverse Targetable Genomic Alterations, Including Kinase Fusions. *Oncologist* **2017**, *22*, 255-263, doi:10.1634/theoncologist.2016-0279.
41. Hardee, S.; Prasad, M.L.; Hui, P.; Dinauer, C.A.; Morotti, R.A. Pathologic Characteristics, Natural History, and Prognostic Implications of BRAF(V600E) Mutation in Pediatric Papillary Thyroid Carcinoma. *Pediatr Dev Pathol* **2017**, *20*, 206-212, doi:10.1177/1093526616689628.
42. Franco, A.T.; Labourier, E.; Ablordeppey, K.K.; Surrey, L.F.; Mostoufi-Moab, S.; Isaza, A.; Adzick, N.S.; Kazahaya, K.; Kumar, G.; Bauer, A.J. miRNA expression can classify pediatric thyroid lesions and increases the diagnostic yield of mutation testing. *Pediatr Blood Cancer* **2020**, *67*, e28276, doi:10.1002/pbc.28276.
43. Klugbauer, S.; Lengfelder, E.; Demidchik, E.P.; Rabes, H.M. High prevalence of RET rearrangement in thyroid tumors of children from Belarus after the Chernobyl reactor accident. *Oncogene* **1995**, *11*, 2459-2467.
44. Nikiforov, Y.E.; Rowland, J.M.; Bove, K.E.; Monforte-Munoz, H.; Fagin, J.A. Distinct pattern of ret oncogene rearrangements in morphological variants of radiation-induced and sporadic thyroid papillary carcinomas in children. *Cancer Res* **1997**, *57*, 1690-1694.
45. Smida, J.; Salassidis, K.; Hieber, L.; Zitzelsberger, H.; Kellerer, A.M.; Demidchik, E.P.; Negele, T.; Spelsberg, F.; Lengfelder, E.; Werner, M., et al. Distinct frequency of ret rearrangements in papillary thyroid carcinomas of children and adults from Belarus. *Int J Cancer* **1999**, *80*, 32-38, doi:10.1002/(sici)1097-0215(19990105)80:1<32::aid-ijc7>3.0.co;2-l.
46. Santoro, M.; Thomas, G.A.; Vecchio, G.; Williams, G.H.; Fusco, A.; Chiappetta, G.; Pozcharskaya, V.; Bogdanova, T.I.; Demidchik, E.P.; Cherstvoy, E.D., et al. Gene rearrangement and Chernobyl related thyroid cancers. *Br J Cancer* **2000**, *82*, 315-322, doi:10.1054/bjoc.1999.0921.
47. Pisarchik, A.V.; Ermak, G.; Fomicheva, V.; Kartel, N.A.; Figge, J. The ret/PTC1 rearrangement is a common feature of Chernobyl-associated papillary thyroid carcinomas from Belarus. *Thyroid* **1998**, *8*, 133-139, doi:10.1089/thy.1998.8.133.
48. Pisarchik, A.V.; Ermak, G.; Demidchik, E.P.; Mikhalevich, L.S.; Kartel, N.A.; Figge, J. Low prevalence of the ret/PTC3r1 rearrangement in a series of papillary thyroid carcinomas presenting in Belarus ten years post-Chernobyl. *Thyroid* **1998**, *8*, 1003-1008, doi:10.1089/thy.1998.8.1003.
49. Thomas, G.A.; Bunnell, H.; Cook, H.A.; Williams, E.D.; Nerovnya, A.; Cherstvoy, E.D.; Tronko, N.D.; Bogdanova, T.I.; Chiappetta, G.; Viglietto, G., et al. High

prevalence of RET/PTC rearrangements in Ukrainian and Belarussian post-Chernobyl thyroid papillary carcinomas: a strong correlation between RET/PTC3 and the solid-follicular variant. *J Clin Endocrinol Metab* **1999**, *84*, 4232-4238, doi:10.1210/jcem.84.11.6129.
50. Rabes, H.M.; Demidchik, E.P.; Sidorow, J.D.; Lengfelder, E.; Beimfohr, C.; Hoelzel, D.; Klugbauer, S. Pattern of radiation-induced RET and NTRK1 rearrangements in 191 post-chernobyl papillary thyroid carcinomas: biological, phenotypic, and clinical implications. *Clin Cancer Res* **2000**, *6*, 1093-1103.
51. Elisei, R.; Romei, C.; Vorontsova, T.; Cosci, B.; Veremeychik, V.; Kuchinskaya, E.; Basolo, F.; Demidchik, E.P.; Miccoli, P.; Pinchera, A., et al. RET/PTC rearrangements in thyroid nodules: studies in irradiated and not irradiated, malignant and benign thyroid lesions in children and adults. *J Clin Endocrinol Metab* **2001**, *86*, 3211-3216, doi:10.1210/jcem.86.7.7678.
52. Detours, V.; Wattel, S.; Venet, D.; Hutsebaut, N.; Bogdanova, T.; Tronko, M.D.; Dumont, J.E.; Franc, B.; Thomas, G.; Maenhaut, C. Absence of a specific radiation signature in post-Chernobyl thyroid cancers. *Br J Cancer* **2005**, *92*, 1545-1552, doi:10.1038/sj.bjc.6602521.
53. Bounacer, A.; Wicker, R.; Caillou, B.; Cailleux, A.F.; Sarasin, A.; Schlumberger, M.; Suarez, H.G. High prevalence of activating ret proto-oncogene rearrangements, in thyroid tumors from patients who had received external radiation. *Oncogene* **1997**, *15*, 1263-1273, doi:10.1038/sj.onc.1200206.
54. Basolo, F.; Giannini, R.; Monaco, C.; Melillo, R.M.; Carlomagno, F.; Pancrazi, M.; Salvatore, G.; Chiappetta, G.; Pacini, F.; Elisei, R., et al. Potent mitogenicity of the RET/PTC3 oncogene correlates with its prevalence in tall-cell variant of papillary thyroid carcinoma. *Am J Pathol* **2002**, *160*, 247-254, doi:10.1016/S0002-9440(10)64368-4.
55. Bongarzone, I.; Fugazzola, L.; Vigneri, P.; Mariani, L.; Mondellini, P.; Pacini, F.; Basolo, F.; Pinchera, A.; Pilotti, S.; Pierotti, M.A. Age-related activation of the tyrosine kinase receptor protooncogenes RET and NTRK1 in papillary thyroid carcinoma. *J Clin Endocrinol Metab* **1996**, *81*, 2006-2009, doi:10.1210/jcem.81.5.8626874.
56. Wang, Y.L.; Zhang, R.M.; Luo, Z.W.; Wu, Y.; Du, X.; Wang, Z.Y.; Zhu, Y.X.; Li, D.S.; Ji, Q.H. High frequency of level II-V lymph node involvement in RET/PTC positive papillary thyroid carcinoma. *Eur J Surg Oncol* **2008**, *34*, 77-81, doi:10.1016/j.ejso.2007.08.012.
57. Lam, K.Y.; Lo, C.Y.; Leung, P.S. High prevalence of RET proto-oncogene activation (RET/PTC) in papillary thyroid carcinomas. *Eur J Endocrinol* **2002**, *147*, 741-745.
58. Nakazawa, T.; Kondo, T.; Kobayashi, Y.; Takamura, N.; Murata, S.; Kameyama, K.; Muramatsu, A.; Ito, K.; Kobayashi, M.; Katoh, R. RET gene rearrangements

(RET/PTC1 and RET/PTC3) in papillary thyroid carcinomas from an iodine-rich country (Japan). *Cancer* **2005**, *104*, 943-951, doi:10.1002/cncr.21270.

59. Motomura, T.; Nikiforov, Y.E.; Namba, H.; Ashizawa, K.; Nagataki, S.; Yamashita, S.; Fagin, J.A. ret rearrangements in Japanese pediatric and adult papillary thyroid cancers. *Thyroid* **1998**, *8*, 485-489, doi:10.1089/thy.1998.8.485.
60. Rhoden, K.J.; Johnson, C.; Brandao, G.; Howe, J.G.; Smith, B.R.; Tallini, G. Real-time quantitative RT-PCR identifies distinct c-RET, RET/PTC1 and RET/PTC3 expression patterns in papillary thyroid carcinoma. *Lab Invest* **2004**, *84*, 1557-1570, doi:10.1038/labinvest.3700198.
61. Fenton, C.L.; Lukes, Y.; Nicholson, D.; Dinauer, C.A.; Francis, G.L.; Tuttle, R.M. The ret/PTC mutations are common in sporadic papillary thyroid carcinoma of children and young adults. *J Clin Endocrinol Metab* **2000**, *85*, 1170-1175, doi:10.1210/jcem.85.3.6472.
62. Leeman-Neill, R.J.; Kelly, L.M.; Liu, P.; Brenner, A.V.; Little, M.P.; Bogdanova, T.I.; Evdokimova, V.N.; Hatch, M.; Zurnadzy, L.Y.; Nikiforova, M.N., et al. ETV6-NTRK3 is a common chromosomal rearrangement in radiation-associated thyroid cancer. *Cancer* **2014**, *120*, 799-807, doi:10.1002/cncr.28484.
63. Picarsic, J.L.; Buryk, M.A.; Ozolek, J.; Ranganathan, S.; Monaco, S.E.; Simons, J.P.; Witchel, S.F.; Gurtunca, N.; Joyce, J.; Zhong, S., et al. Molecular Characterization of Sporadic Pediatric Thyroid Carcinoma with the DNA/RNA ThyroSeq v2 Next-Generation Sequencing Assay. *Pediatr Dev Pathol* **2016**, *19*, 115-122, doi:10.2350/15-07-1667-OA.1.
64. Prasad, M.L.; Vyas, M.; Horne, M.J.; Virk, R.K.; Morotti, R.; Liu, Z.; Tallini, G.; Nikiforova, M.N.; Christison-Lagay, E.R.; Udelsman, R., et al. NTRK fusion oncogenes in pediatric papillary thyroid carcinoma in northeast United States. *Cancer* **2016**, *122*, 1097-1107, doi:10.1002/cncr.29887.
65. Nikiforov, Y.E.; Nikiforova, M.N.; Gnepp, D.R.; Fagin, J.A. Prevalence of mutations of ras and p53 in benign and malignant thyroid tumors from children exposed to radiation after the Chernobyl nuclear accident. *Oncogene* **1996**, *13*, 687-693.
66. Suchy, B.; Waldmann, V.; Klugbauer, S.; Rabes, H.M. Absence of RAS and p53 mutations in thyroid carcinomas of children after Chernobyl in contrast to adult thyroid tumours. *Br J Cancer* **1998**, *77*, 952-955, doi:10.1038/bjc.1998.157.
67. Pauws, E.; Tummers, R.F.; Ris-Stalpers, C.; de Vijlder, J.J.; Voute, T. Absence of activating mutations in ras and gsp oncogenes in a cohort of nine patients with sporadic pediatric thyroid tumors. *Med Pediatr Oncol* **2001**, *36*, 630-634, doi:10.1002/mpo.1140.
68. Fenton, C.; Anderson, J.; Lukes, Y.; Dinauer, C.A.; Tuttle, R.M.; Francis, G.L. Ras mutations are uncommon in sporadic thyroid cancer in children and young adults. *J Endocrinol Invest* **1999**, *22*, 781-789, doi:10.1007/BF03343644.
